# Experimental data of charring of timber elements under natural fire: Full-scale fire testing of timber structures using the room corner test method

**DOI:** 10.1016/j.dib.2025.112410

**Published:** 2025-12-22

**Authors:** Jakub Šejna, Dominik Štraus, Marie Křišťanová, Simona Rušarová, Kamila Cábová, Robert Pečenko, Tomaž Hozjan, František Wald

**Affiliations:** a Department of Steel and Timber Structures, Faculty of Civil Engineering, Czech Technical University in Prague, Thákurova 7, 166 29 Prague 6, Czech Republic; b Department of Mechanics, Faculty of Civil Engineering and Geodesy, University of Ljubljana, Slovenia

**Keywords:** Fire resistance, Wood charring, Heat transfer, Full-scale experiments, Timber structures, Room corner test, Temperature measurement, Natural fire exposure

## Abstract

The paper presents results of an experimental investigation of charring of full-scale timber elements exposed to natural fire conditions. Fire experiments were conducted in the Room Corner Test facility, in which natural local fire scenarios were simulated to assess realistic charring rate and thermal response of timber structures. The analysis of experimental data evaluates the effects of timber orientation relative to the fire source, heat transfer dynamics, and other duration of fire exposure. The results demonstrate that charring rate is influenced not only by direct flame exposure but also by radiative and convective heat fluxes, leading to non-uniform charring depths along the height and across different sides of timber elements. The provided data include detailed temperature measurements at multiple depths of timber elements, high-resolution photographic and thermographic documentation to demonstrate charred layer formation, pyrolysis depth, and material loss. The dataset, made available for further research, provides a valuable foundation for numerical fire model validation and supports the development of probabilistic approaches for predicting variability of charring.

Specifications Table

**Table:** 

Subject	Engineering & Materials science
Specific subject area	Experimental fire testing of timber structures to determine charring behavior and temperature profiles under natural fire conditions.
Type of data	Table; Figure; Graph; Image; Chart Raw; Processed
Data collection	Full-scale fire tests were conducted in the fire testing laboratory of the Czech Technical University in Prague using the Room Corner Test configuration. Timber columns were exposed to natural fire conditions. Temperature data were collected with K-type thermocouples (Ø0.5 mm, MAVIS, Czech Republic) embedded within and behind the char layer. Measurements were recorded every 3 s using a multi-channel data acquisition system, ensuring high temporal resolution for subsequent thermal analysis.
Data source location	FireLAB, University Centre for Energy Efficient Buildings (UCEEB), Czech Technical University in Prague, Třinecká 1024, 273 43 Buštěhrad, Czech Republic.
Data accessibility	Repository name: Zenodo Data identification number doi:10.5281/zenodo.17312555 Direct URL to data: https://doi.org/10.5281/zenodo.17312555 Instructions for accessing these data: The dataset is openly available on the Zenodo repository under the Creative Commons Attribution (CC BY 4.0) license. All files can be freely downloaded without restrictions or registration. The repository contains raw measurement data, processed graphs, and complete photographic documentation associated with the Room Corner Test experiments described in this article. All reviewers and editors can access it without registration.
Related research article	none

## 1 Value of the Data

• These data provide a unique full-scale natural fire dataset experimental record of charring behavior of timber elements under natural fire exposure, obtained using the standardized Room Corner Test (RCT) configuration. Such comprehensive datasets are rare, as most previous studies are based on small-scale or standard fire conditions.
• The dataset offers high-resolution temperature measurements from multiple depths and positions within the timber members, recorded every 3 s, enabling detailed thermal analysis and model validation for both transient and steady-state heat transfer conditions.
• The data can be directly used for validation of numerical and analytical models of charring and pyrolysis, including coupled heat–mass transfer models such as PyCiF. Researchers can test and calibrate their predictive algorithms against measured temperature–depth profiles and final charring geometries.
• The dataset includes systematic photographic documentation, covering both the preparation and the post-fire cross-section analysis of all specimens. This allows other researchers to evaluate measurement consistency, visualize the progression of charring, and conduct comparative optical or geometric analyses.
• The availability of raw and processed data in open format facilitates reproducibility and data reuse for sensitivity analyses, uncertainty quantification, and probabilistic modelling of wood charring under variable natural fire scenarios.

## 2 Background

The behaviour of timber under fire conditions represents a fundamental challenge in fire safety and structural engineering. Conventional approaches for determining char layer development rely mainly on empirical models derived from standard fire exposures. However, these models do not fully capture the variability of natural fire conditions, where heat flux, temperature evolution, and fire dynamics differ significantly, and where factors such as element orientation play a crucial role. With the growing use of timber in modern architecture, including multi-storey and hybrid buildings, a more detailed understanding of charring mechanisms and their influence on structural fire resistance is essential. The dataset originates from an experimental study investigating the charring behaviour of timber columns under natural fire conditions using the Room Corner Test (RCT) method. The collected data provide a comprehensive foundation for validating numerical models and improving the accuracy of analytical predictions of timber performance in natural fire scenarios.

## 3 Data Description

### 3.1 Dataset citation

Šejna, J., Štraus, D., Křišťanová, M., Rušarová, S., Cábová, K., Pečenko, R., Hozjan, T., Wald, F. (2025). Experimental data of Charring of Timber Elements under Natural Fire: Full-Scale Fire Testing of Timber Structures Using the Room Corner Test Method. Zenodo. doi:10.5281/zenodo.17312555.

The dataset comprises eight files organized into two main experimental series, denoted as **OPT_A** and **OPT_B**, each representing a distinct optical depth measurement experiment. The files include both raw measurement data and derived materials such as photographs and processed graphs.

**Table:** 

Filename	Description	File type	Notes
**OPT_A_depth_** **measuring.rar**	Compressed archive containing raw depth-measurement data from experiment series A. Likely includes tabulated numerical results and instrument logs.	.rar archive	Raw data
**OPT_A_TEMP from exp.DAT**	Raw temperature data recorded during experiment A. Contains tabulated temperature–time data.	.DAT text data	Raw data
**OPT_B_depth_** **measuring.rar**	Compressed archive containing raw depth-measurement data from experiment series B.	.rar archive	Raw data
**OPT_B_TEMP from exp.DAT**	Raw temperature data recorded during experiment B. Structure identical to the A-series temperature file.	.DAT text data	Raw data
**OPT_B_photos_** **depth_measuring.rar**	Collection of photographic documentation from experiment B, showing individual stages of the measurement process.	.rar archive	Photo documentation
**OPT_B_graphs_** **depth_measuring.rar**	Processed graphical results from experiment B, likely including plots and visualizations of measured depth profiles.	.rar archive	Processed data
**OPT_B_promo foto full.zip**	Complete photographic documentation from the preparation phase of experiment OPT B, including setup, instrumentation, and test configuration.	.zip archive	Full preparation photo set

The dataset therefore includes both raw experimental data (temperature and depth measurements) and derived materials (graphs and photographs).

In addition, it provides a complete photographic record of the preparation of the OPT B experiments, capturing all relevant stages of equipment setup and experimental arrangement.

Further information on the experimental setup, measurement methods, and instrumentation used to obtain these data is provided in the section EXPERIMENTAL DESIGN, MATERIALS AND METHODS.

## 4 Experimental Design, Materials and Methods

In the past, many studies have focused on testing of timber elements exposed to standard fire [1]. Based on these studies, empirical relationships for the development of the charred layer have been developed. These relationships are dependent on various parameters, such as moisture content, wood density, fire exposure time, and others [[2], [3], [4]]. There are also simple empirical relationships for determining the charring rate given by different standards, such as Eurocode 5 [5]. In this way, engineers are provided with a relatively simple methodology to determine the charred layer of the timber members exposed to standard fire. However, standard fire can rarely describe the development of temperatures in natural fire and consequently the charring rates of timber members exposed to natural fire. Thus, some researchers were also looking at the burning of wood under non-standard fire conditions [[6], [7], [8]].

Despite many experimental studies, empirical charring models are not completely general, since their area of application is narrowed to cases with simple geometry, to the wood species on which the test was conducted, and only to standard fire or specific non-standard fires. Researchers tried to improve the deficiency mentioned using heat [[9], [10], [11], [12]] or coupled heat-mass transfer numerical models [[13], [14], [15], [16], [17], [18]]. Although the range of use of advanced models can be expanded by using heat and heat-mass models, the significant drawback still represents the determination of charring in cases of natural fire, since the charred layer is determined based on the temperature isotherm in a timber. According to the standard [5] the suggested temperature of the charred layer is 300 °C and is validated for standard fire exposure. However, recent research by [[19], [20]] shows that the temperature of the charred layer in a natural fire varies and depends on the type of fire.

One of the recent approach which may be applicable to natural fire conditions also is the numerical model PyCiF [17,20]. PyCiF combines an advanced thermal-mass model with a pyrolysis model. The wood pyrolysis model is derived from cellulose pyrolysis. The second sub-model, the thermal-mass model, is based on the law of conservation of energy and the law of conservation of mass. These describe the heat sharing equations with sorption and transport equations for gases (ambient and pyrolysis) and moisture. Using the PyCiF model, it is possible to determine the depth of charring without having to determine the location of the 300 °C isotherm. Conversely, the output of this model can be the temperature at the boundary of the charred layer. According to the authors, the model applies to both conventional and natural fires and achieves high accuracy when validated with experimental results. This advanced model is based on a detailed description of physiological phenomena in wood during a fire.

Full-scale fire testing plays a crucial role in understanding the behavior of timber elements under natural fire conditions. When exposed to fire, the surface of the timber ignites and burns rapidly, leading to the formation of a charred layer. Beneath this protective layer, unburned wood undergoes pyrolysis, a thermochemical decomposition process occurring in the absence of oxygen. During pyrolysis, various gases, resins, acids, and tars are released, which further contribute to fire growth and influence the thermal environment within the fire compartment. This process not only impacts the rate of fire spread but also alters the heat transfer mechanisms within the enclosure. More recently, full-scale fire tests using the Room Corner Test (RCT) method have gained attention, as they allow for a more realistic assessment of fire behavior in enclosed compartments.

Fire experiments were conducted in RCT at the University Centre for Energy Efficient Buildings of CTU in Prague (UCEEB CTU).

**Fig. 1:**
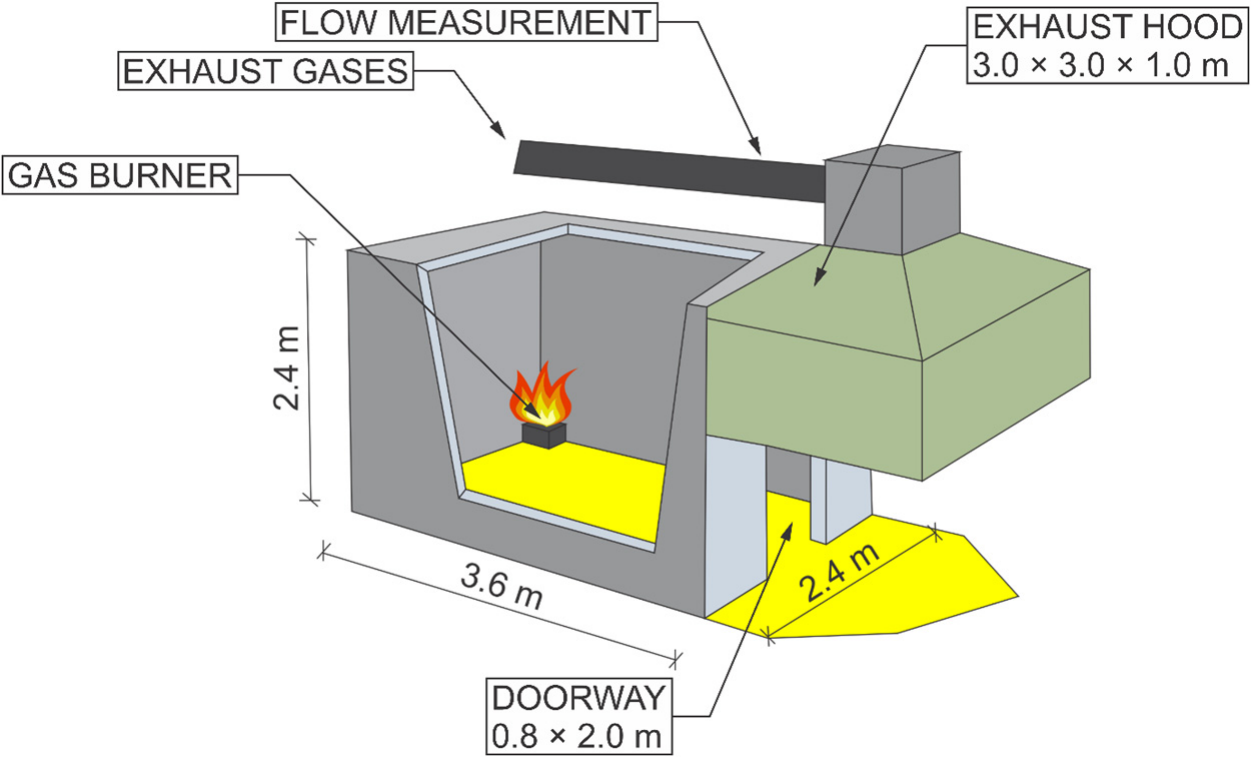
Room corner test in UCEEB laboratory, 3D model.

This RCT and its properties correspond to the EN 14,390 standard. It is a full-scale test facility enclosure with a plan dimension of 3.6 *m* × 2.4 m and a height of 2.4 m. The room is made of aerated concrete blocks. The enclosure has a door opening of 0.8 *m* × 2.0 m and is located on the front wall. A 3 *m* × 3 *m* × 1 m exhaust cone is located adjacent to the room on the front side and is used to exhaust the combustion gases. The smoke is further extracted through an extraction pipe where temperature and flow measurements, gas analysis (O_2_, CO, CO_2_), and optical density measurements are made. The heat source is a gas burner that is placed in a corner of the room. The layout of the room can be seen in Fig. 1.

**Fig. 2:**
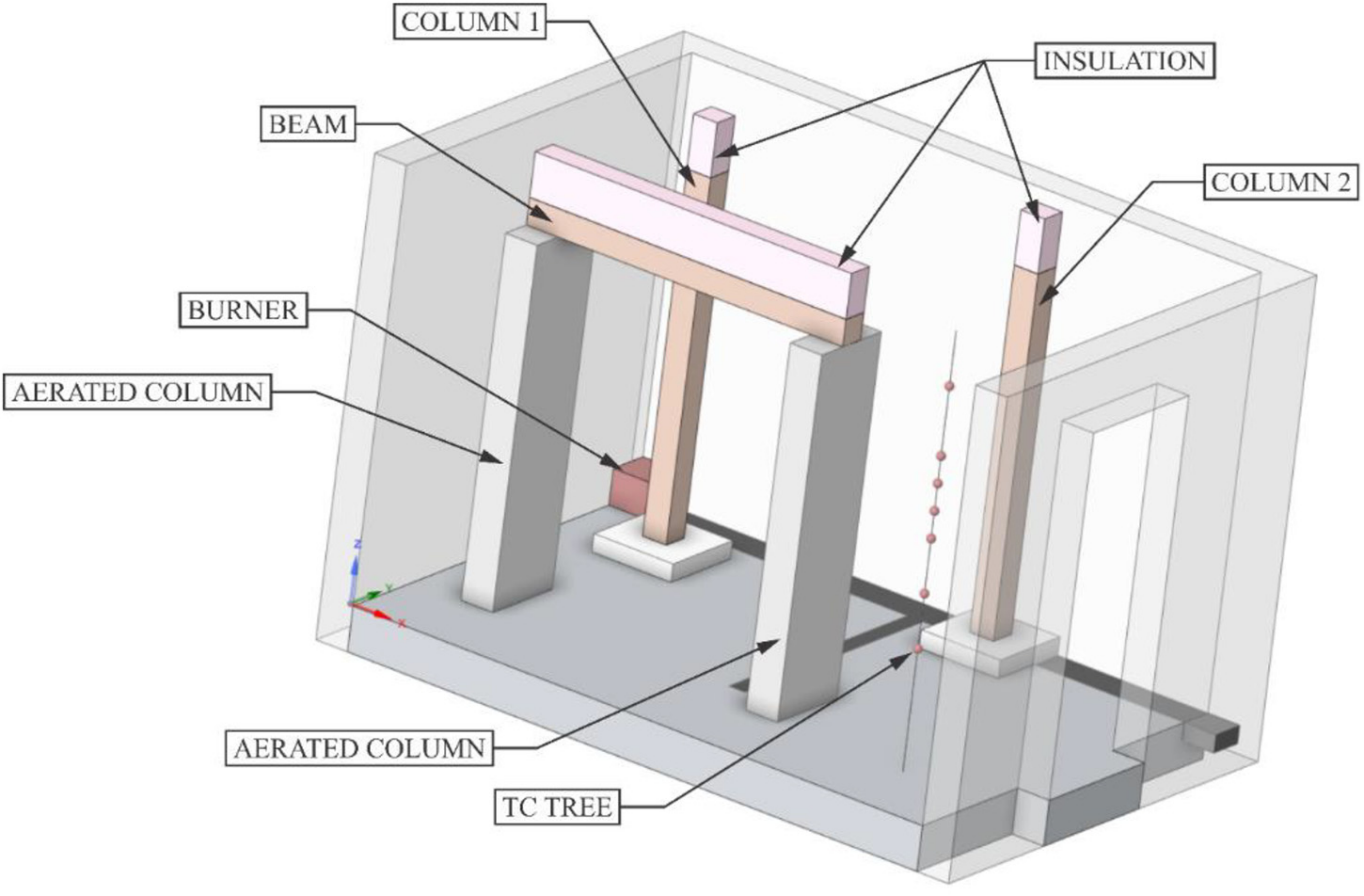
3D scheme of the experiment Option A – two columns and one beam.

**Fig. 3:**
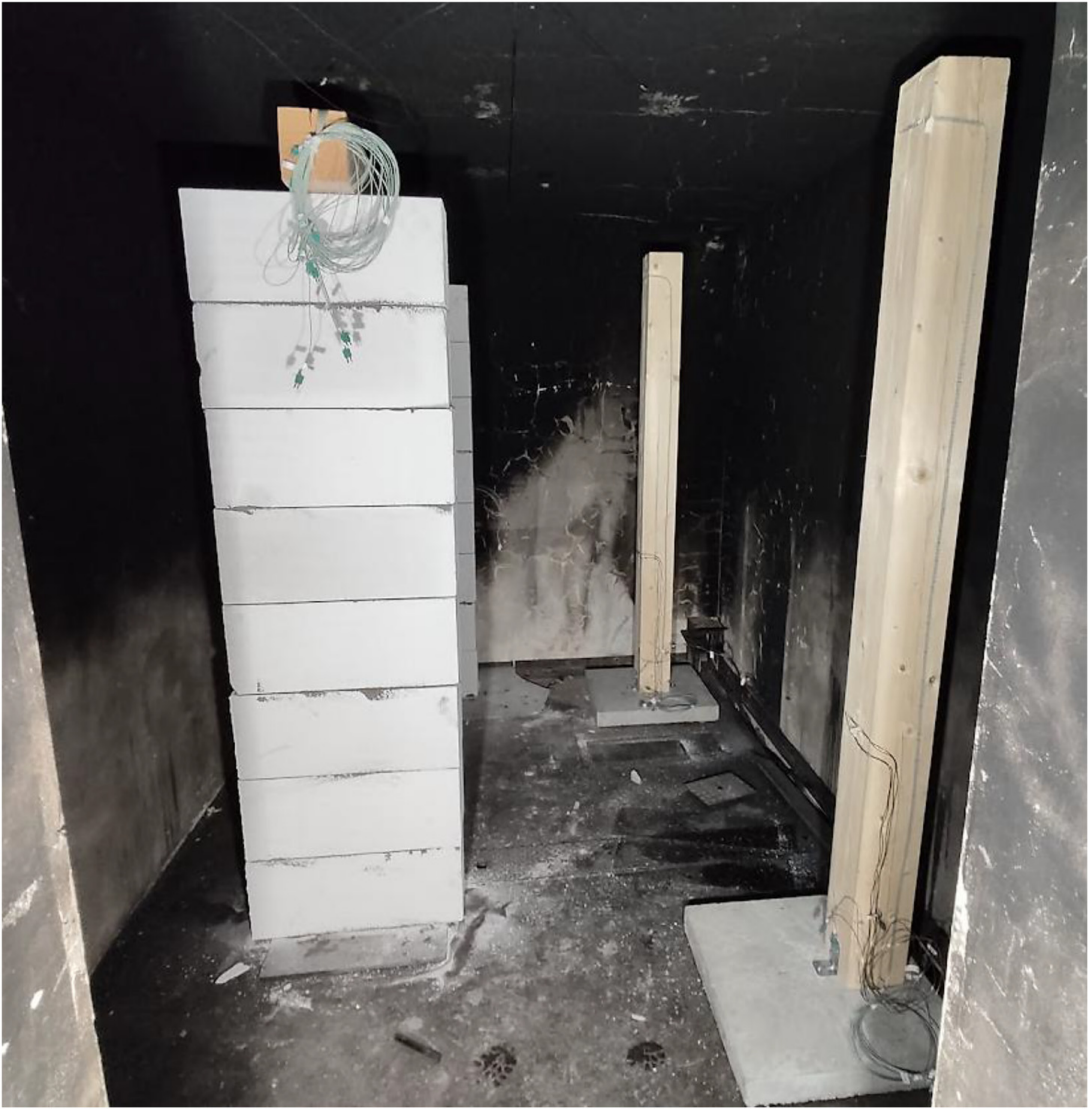
Photo of the experiment Option A – two columns and one beam.

**Fig. 4:**
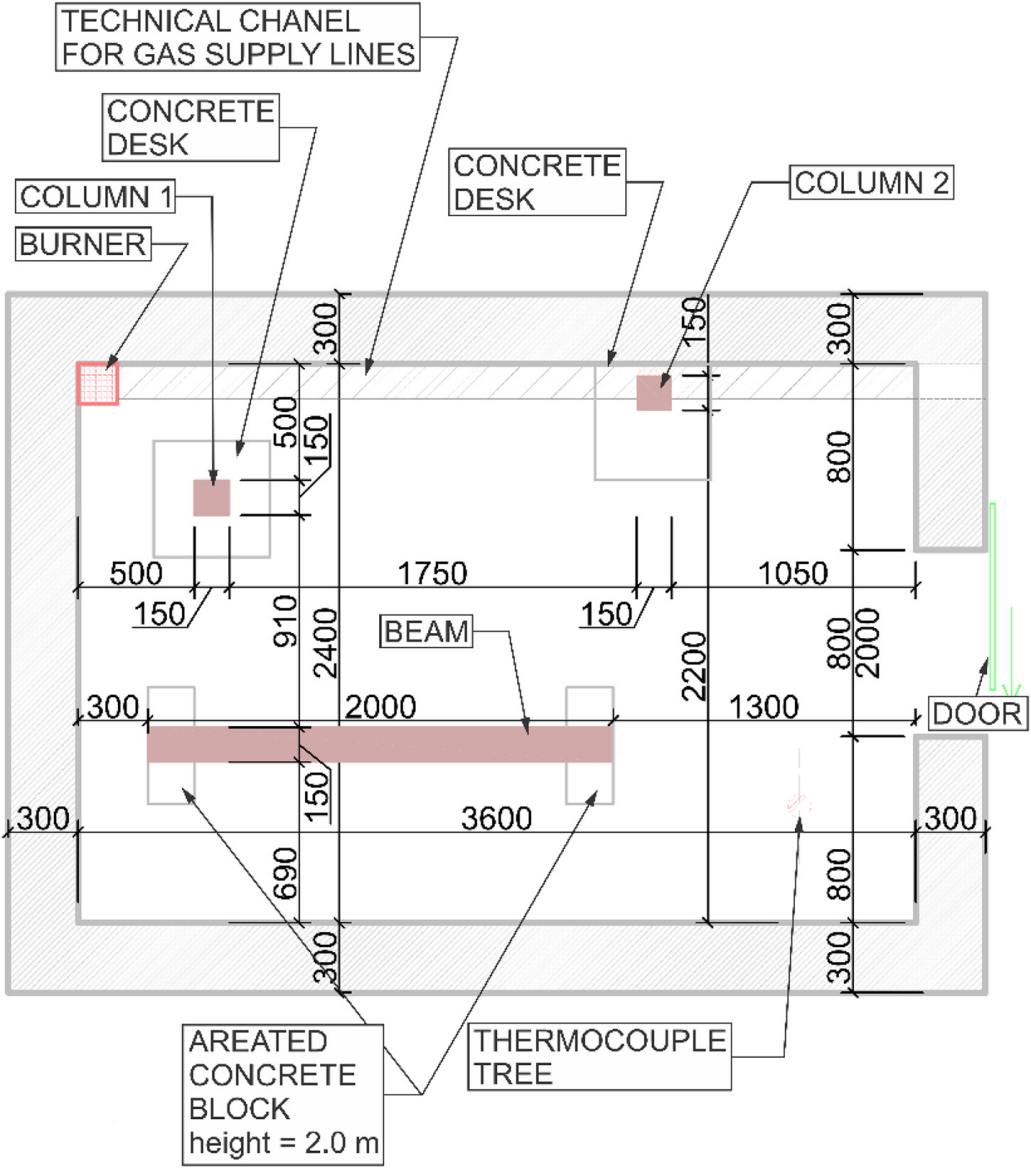
Option A - Floor Plan of experiment.

**Fig. 5:**
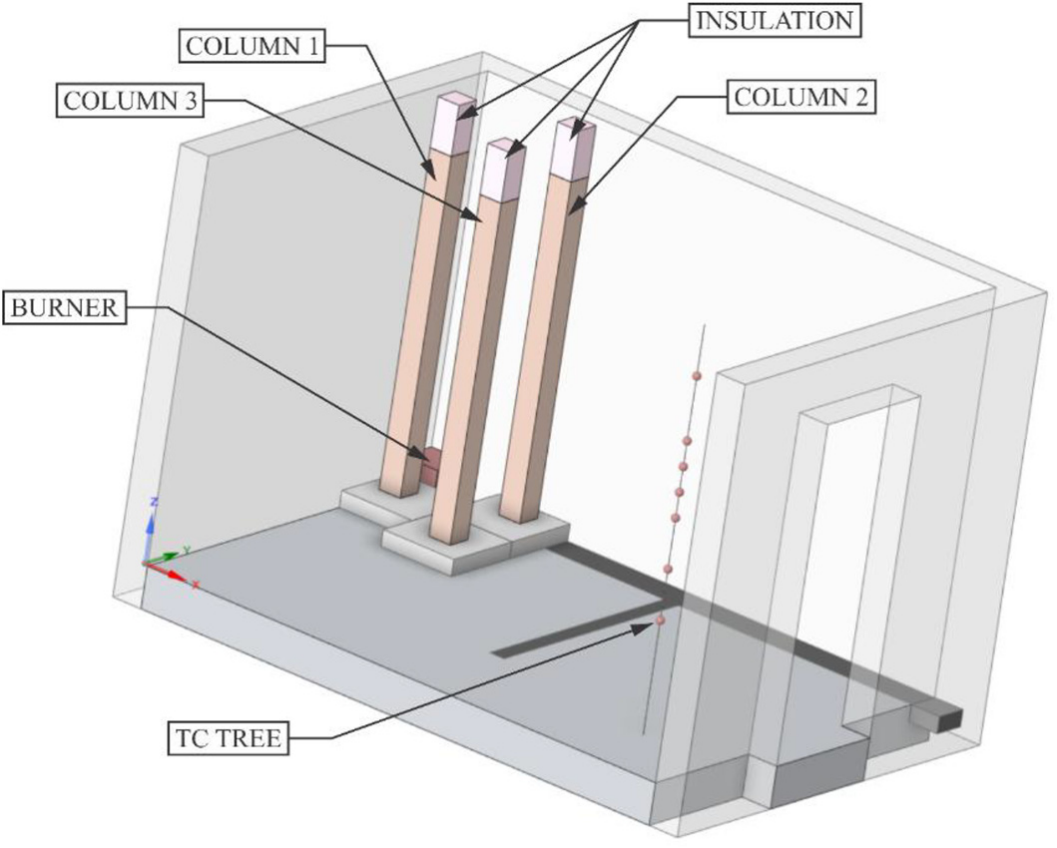
3D scheme of the experiment Option B – tree columns.

**Fig. 6:**
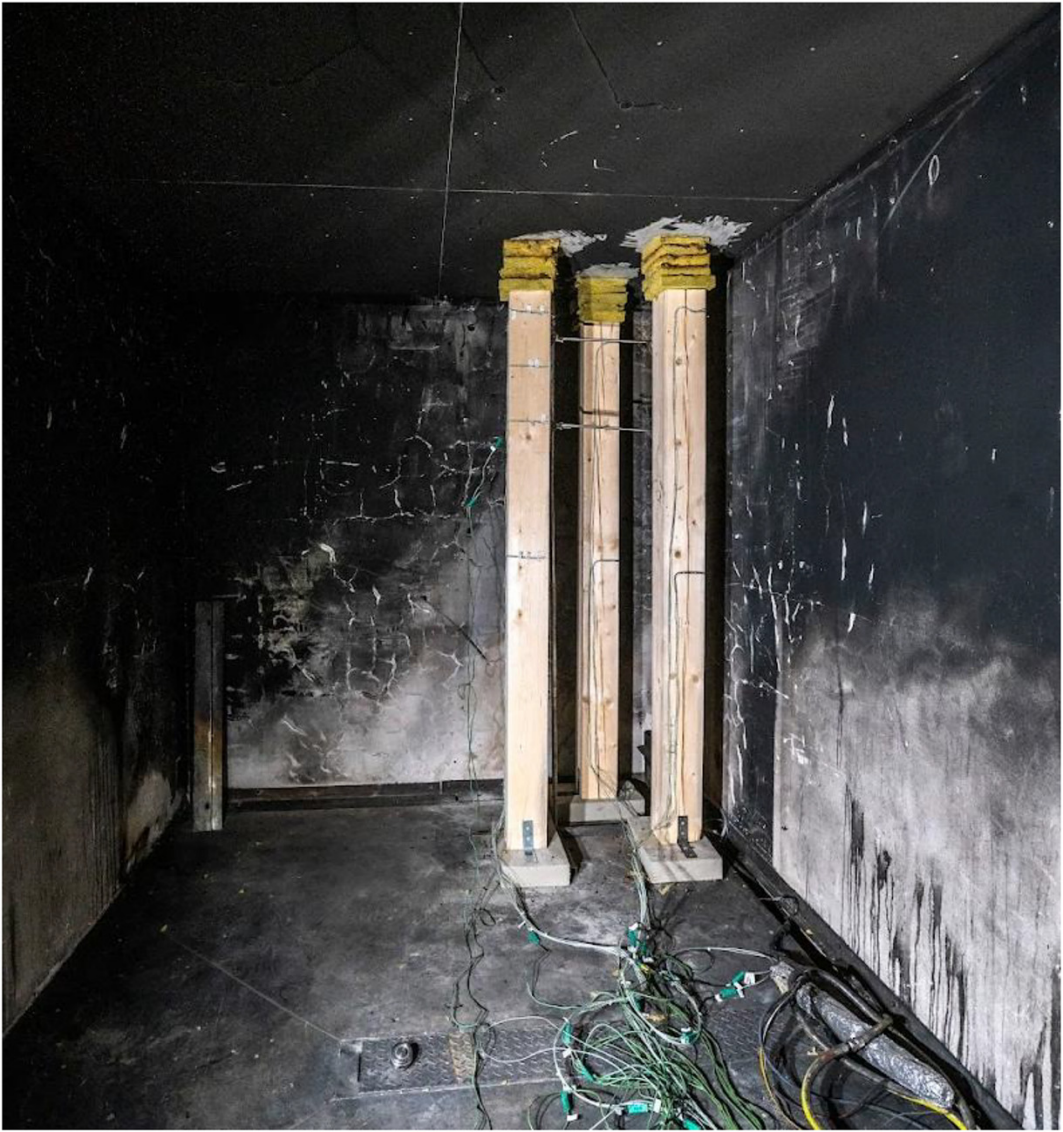
Photo of the experiment Option B – tree columns.

**Fig. 7:**
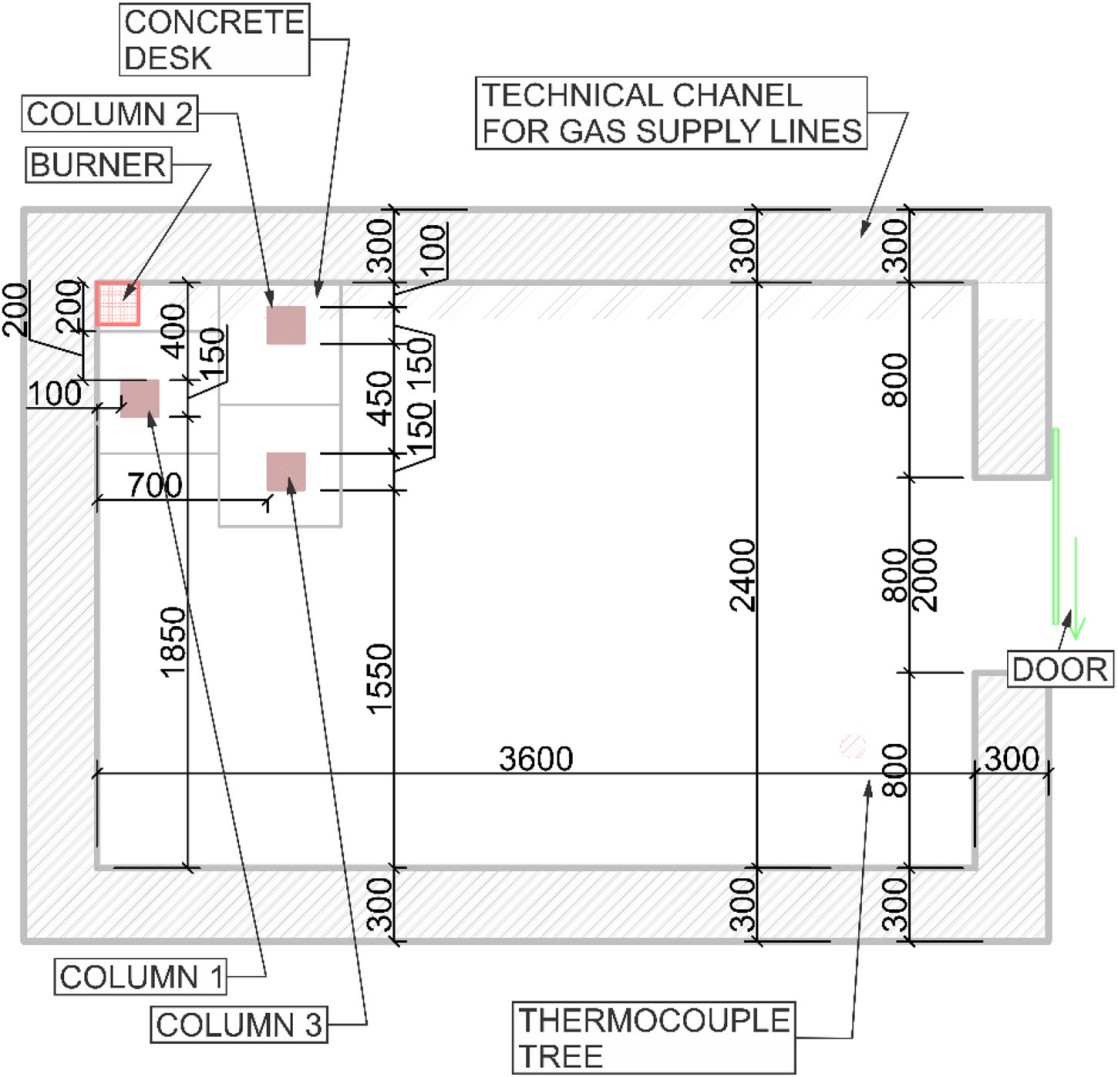
Option B - Floor Plan of experiment.

Two variants (Option A and Option B) of the Room Corner Test (RCT) furnace test were carried out, each incorporating three (Fig. 2) timber members with dimensions of 150 × 150 mm and a length of 2 m. All the members were of spruce wood of strength class C24, initial hudimity 10 %. In Option A, two timber columns and one beam were positioned at a height of 2.0 m above the floor (see Fig. 3, Fig. 4). In this option the beam was supported by two columns made of lightconcrete blocks. In Option B, three columns were placed in close to each other (Fig. 5) (see Fig. 4, Fig. 6). In both configurations, Column 1 was the closest to the burner and consequently experienced the highest thermal exposure. No additional mechanical load was applied to the timber members during the fire tests; all specimens were tested in an unloaded condition (Fig. 7).

A sand propane burner (150 × 150 × 100 mm), located in the corner of the furnace, served as the local fire source in both variants of fire tests. The burner power was identical in both cases, set to 100 kW for the first 10 min, followed by an increase to 300 kW for the remaining 20 min (Fig. 10, Fig. 11). Both experiments were repeated to evaluate the variability of temperature evolution during the tests. Each experiment lasted a total of 30 min. After the test, the timber specimens were extinguished and cooled in a water bath to halt the progression of the charred layer.

**Fig. 8:**
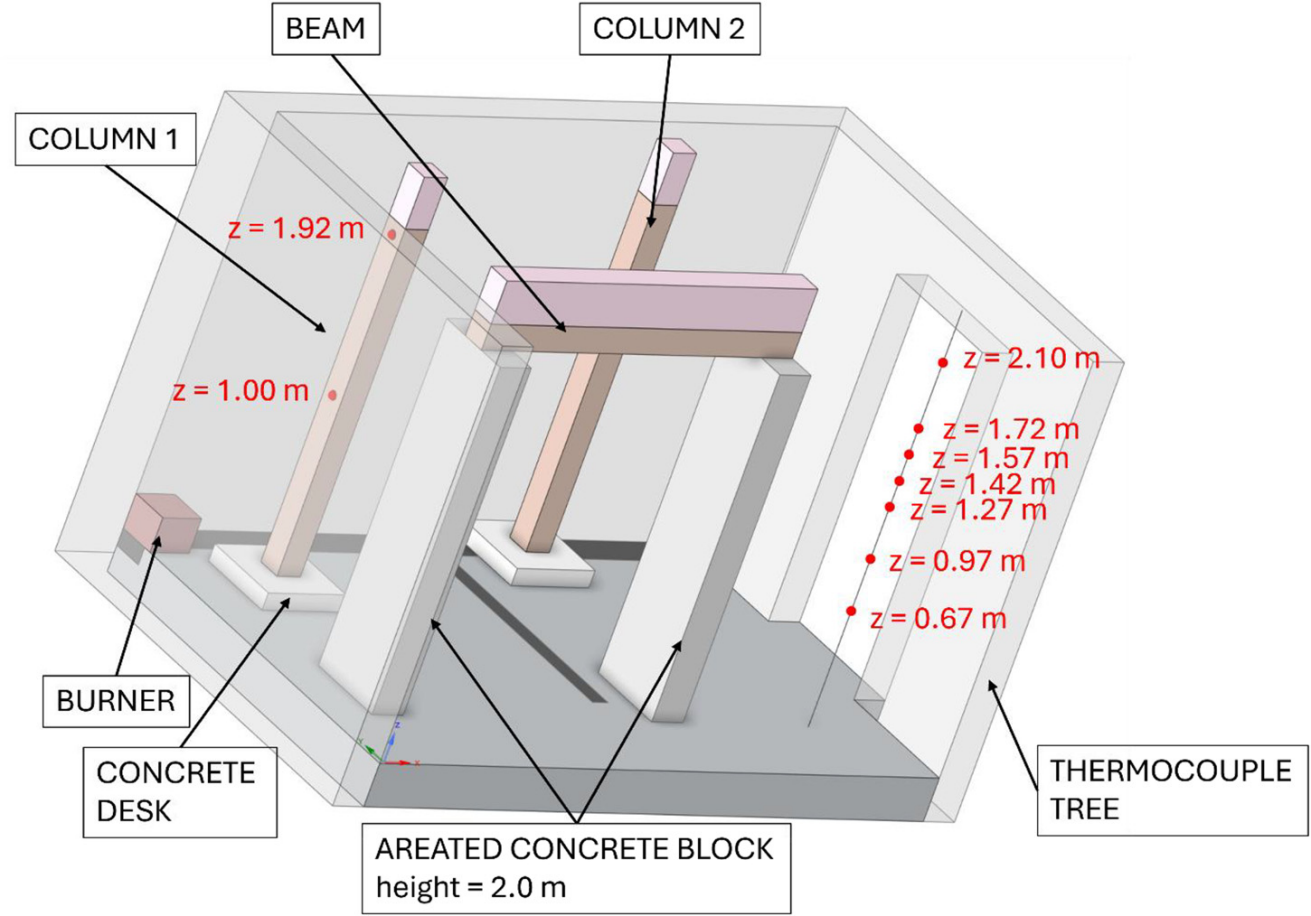
Option A - thermocouple positions.

**Fig. 9:**
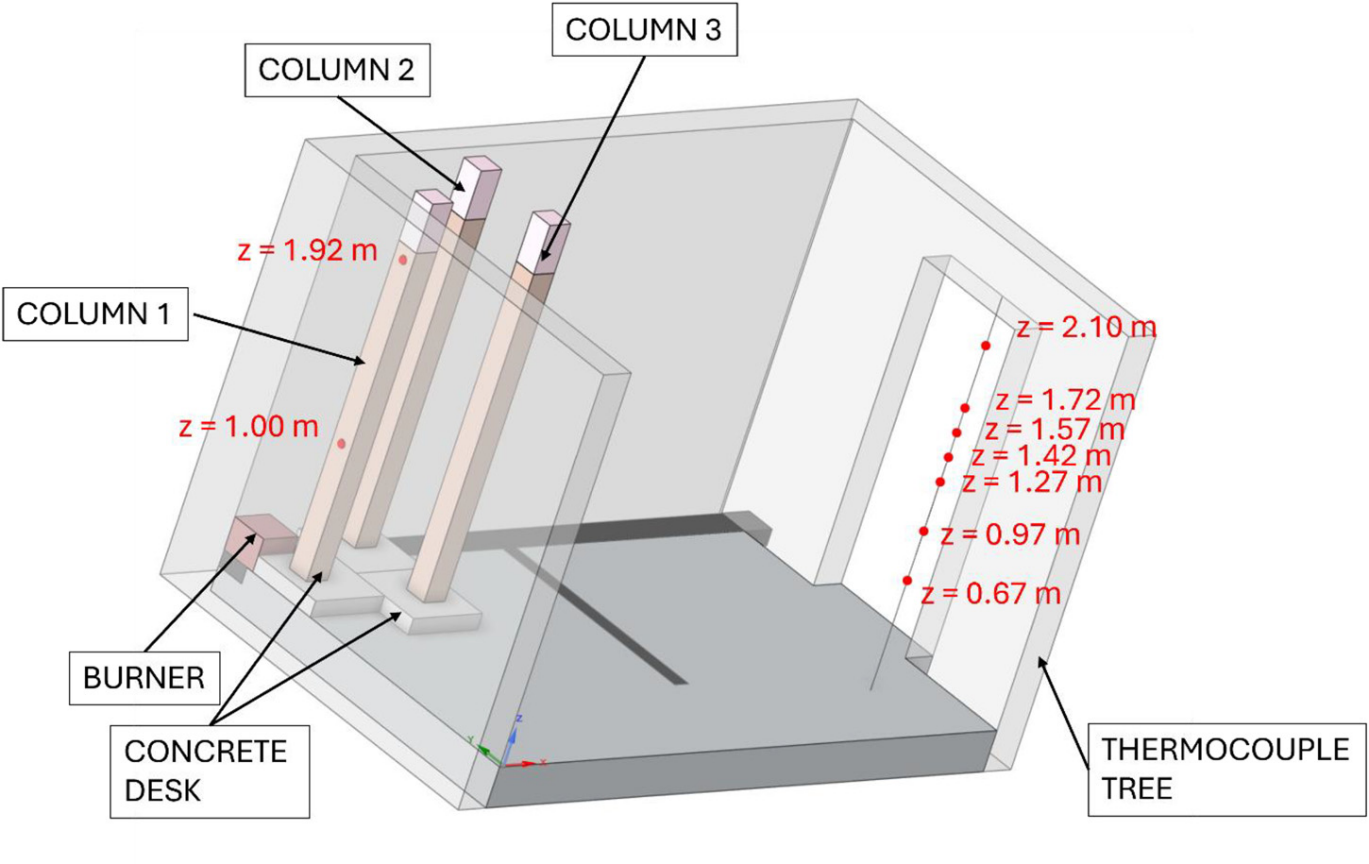
Option B- thermocouple positions.

During the tests, the gas temperature inside the room, temperature on the surface and inside the timber elements were measured, and the smoke produced was recorded. Temperature measurements were obtained using K-type cable thermocouples. Gas temperatures inside the enclosure were recorded using a thermocouple tree (TC tree) and additional thermocouples placed on the ceiling and above the burner, in accordance with RCT fire testing standards. The TC tree recorded temperatures at seven different heights, ranging from 0.67 m to 2.1 m above the floor (see Fig. 8, Fig. 9). The thermocouples embedded in the timber elements were positioned at depths of 5, 15, 25, and 50 mm from the surface and at heights of 1000 mm and 1920 mm from the floor (see Fig. 8 for Option A and Fig. 9) for Option B. To minimize measurement interference and ensure that the thermocouples remained aligned with the relevant layers of wood, all thermocouples were inserted from the side of the timber elements, with the drilling path oriented tangentially to the surface so that the wires would follow the plane of the lamination as closely as possible. The wires were then attached to the least exposed surface of the specimen to ensure that the wiring remained protected from direct heat radiation and flame exposure throughout the experiment. The adiabatic surface temperature of the timber members was measured using plate thermometers (Fig. 12, Fig. 13).

**Fig. 10:**
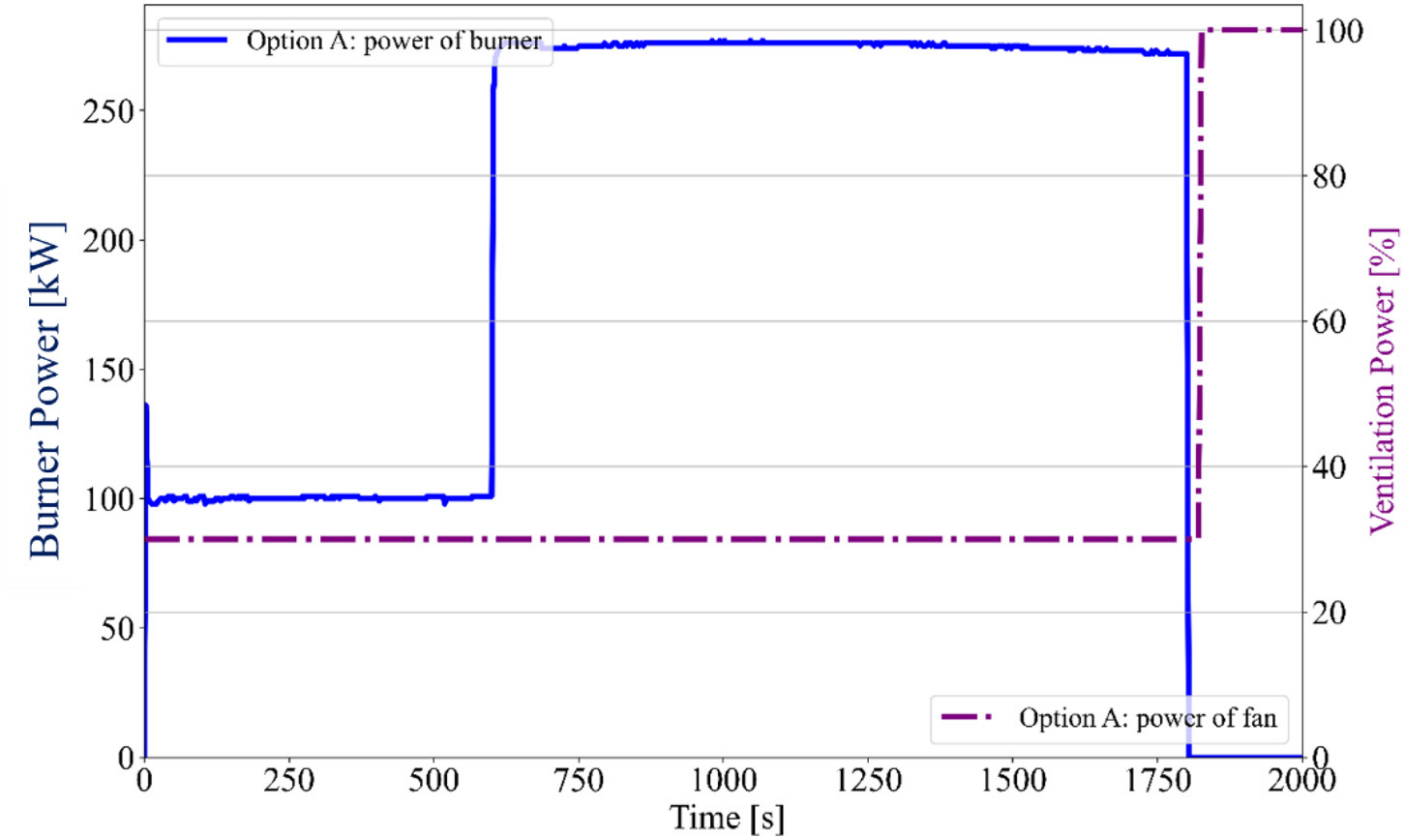
Option A - Burner and Fan power.

**Fig. 11:**
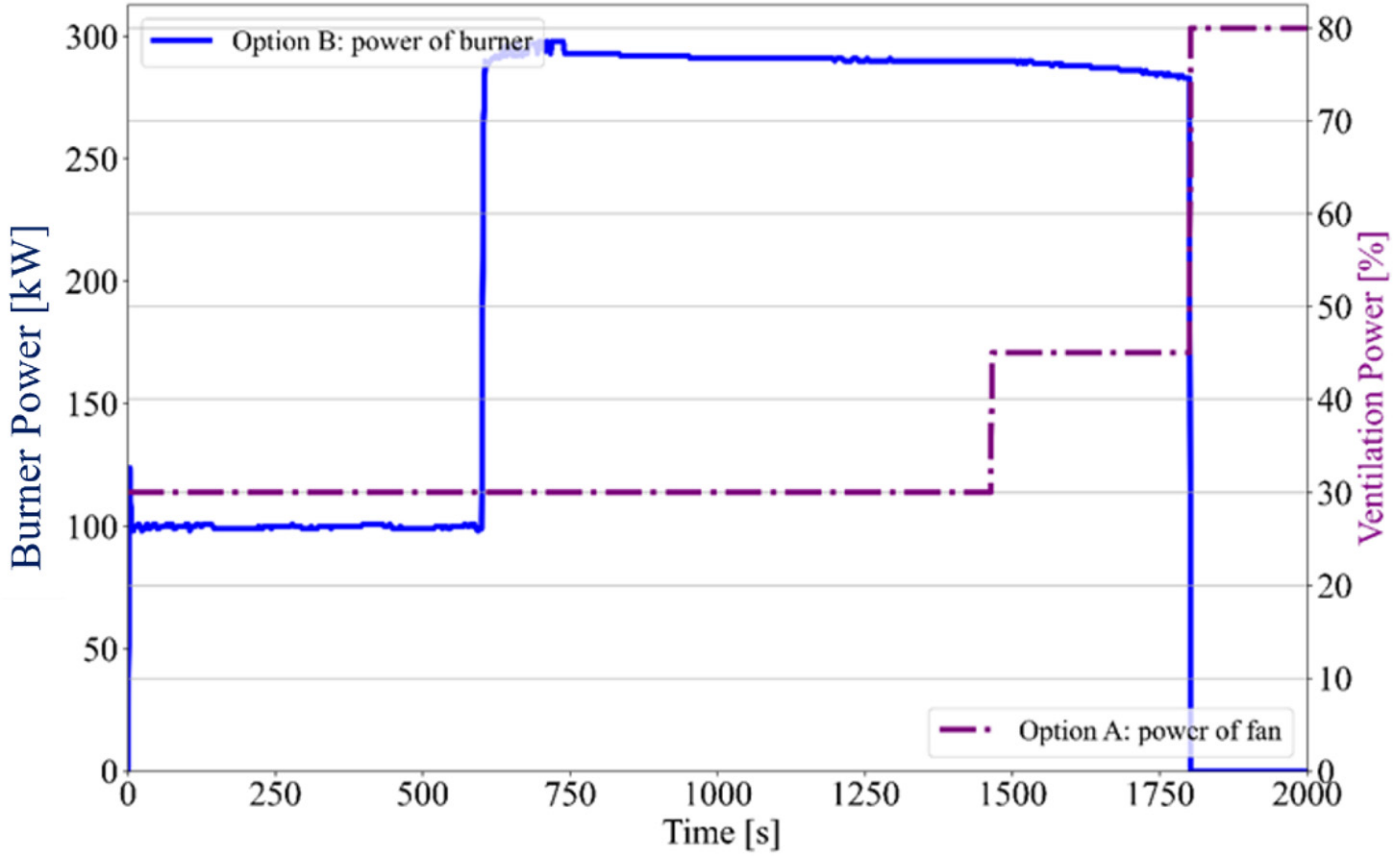
Option B - Burner and Fan power.

**Fig. 12:**
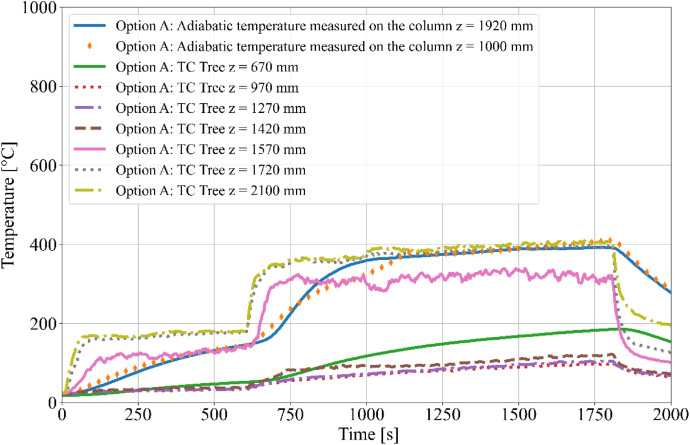
Option A - Temperatures measured on Thermocouple Tree.

**Fig. 13:**
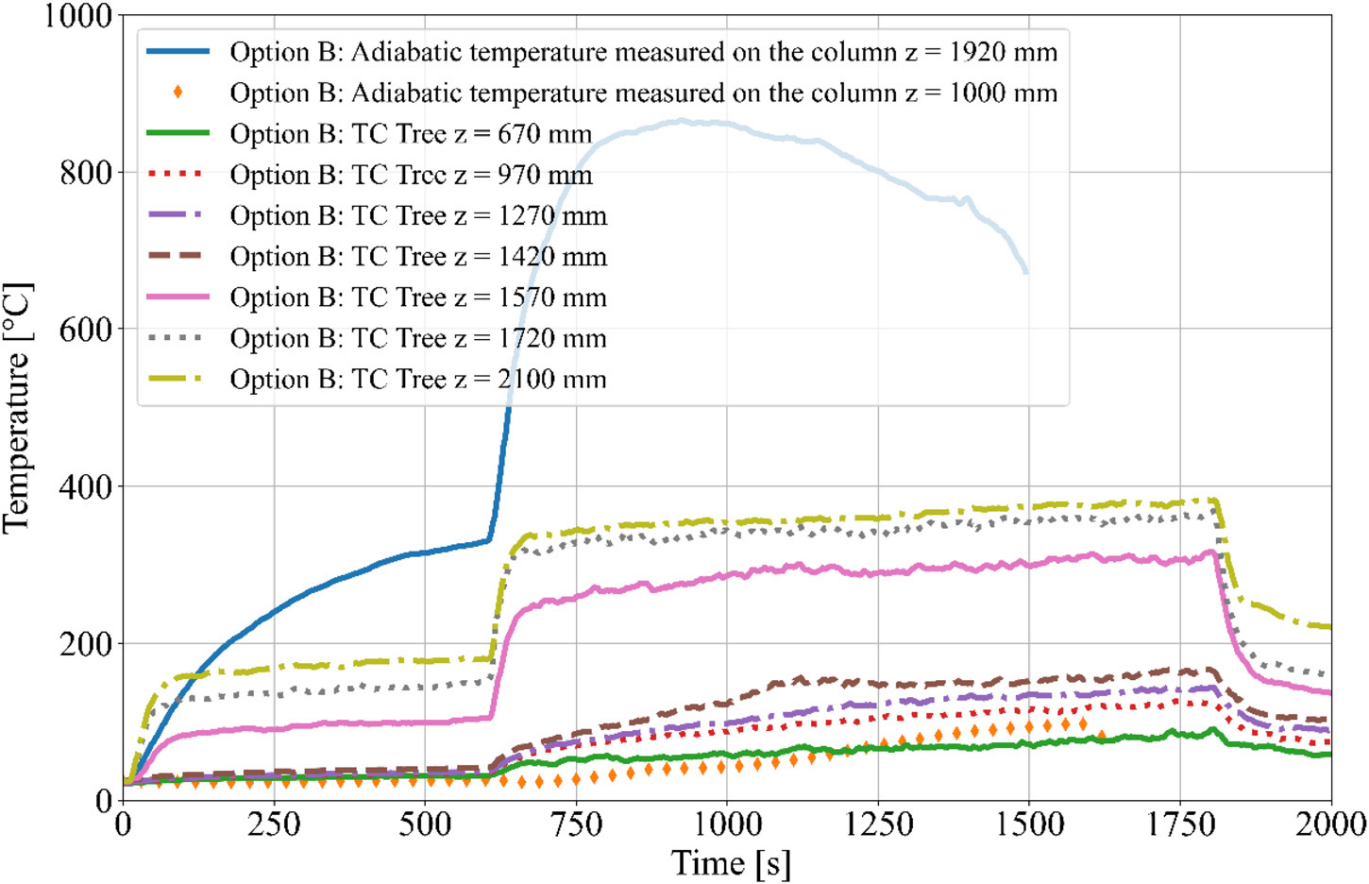
Option B - Temperatures measured on Thermocouple Tree.

**Fig. 14:**
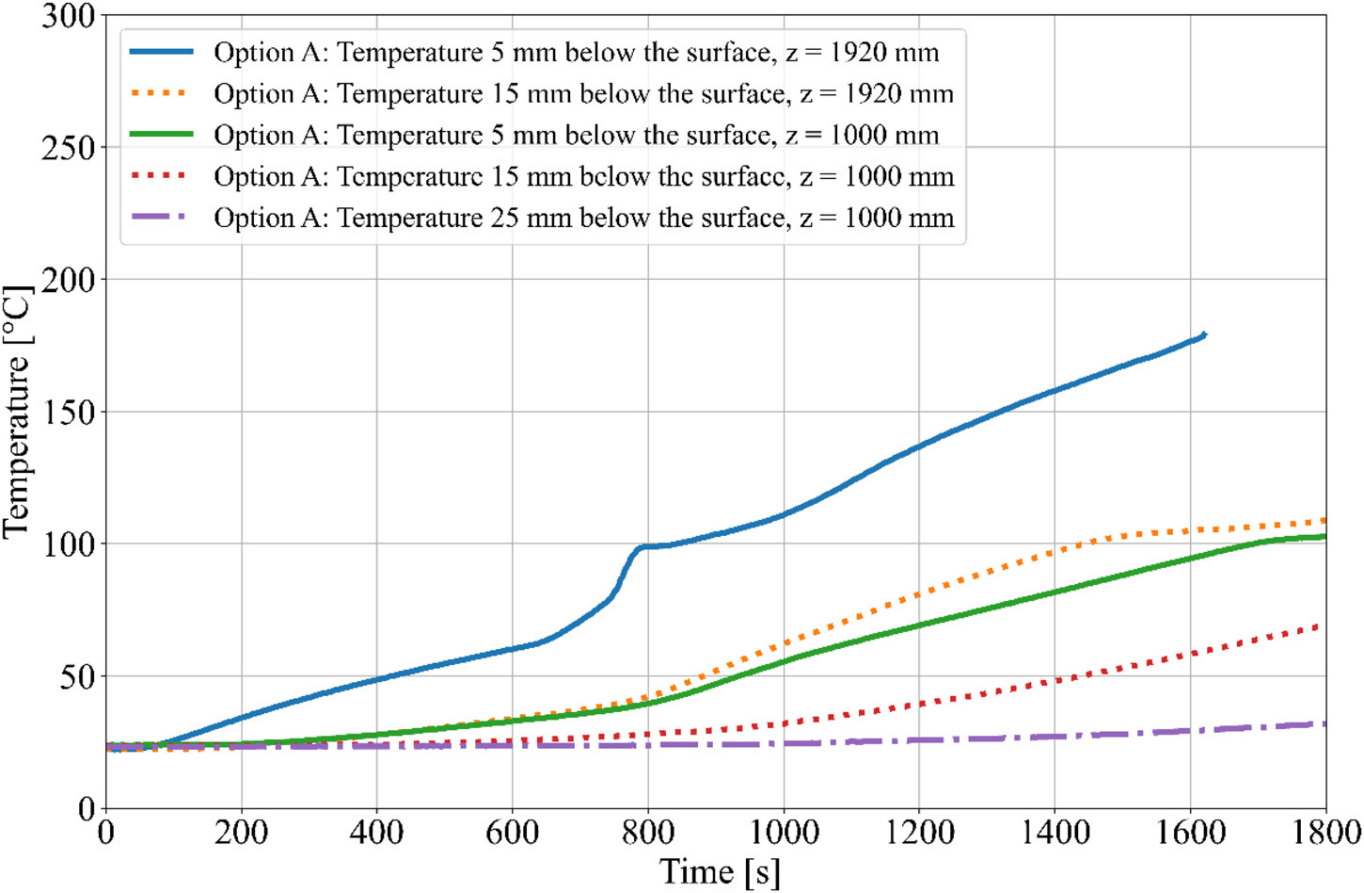
Option A - Column 1: temperature.

**Fig. 15:**
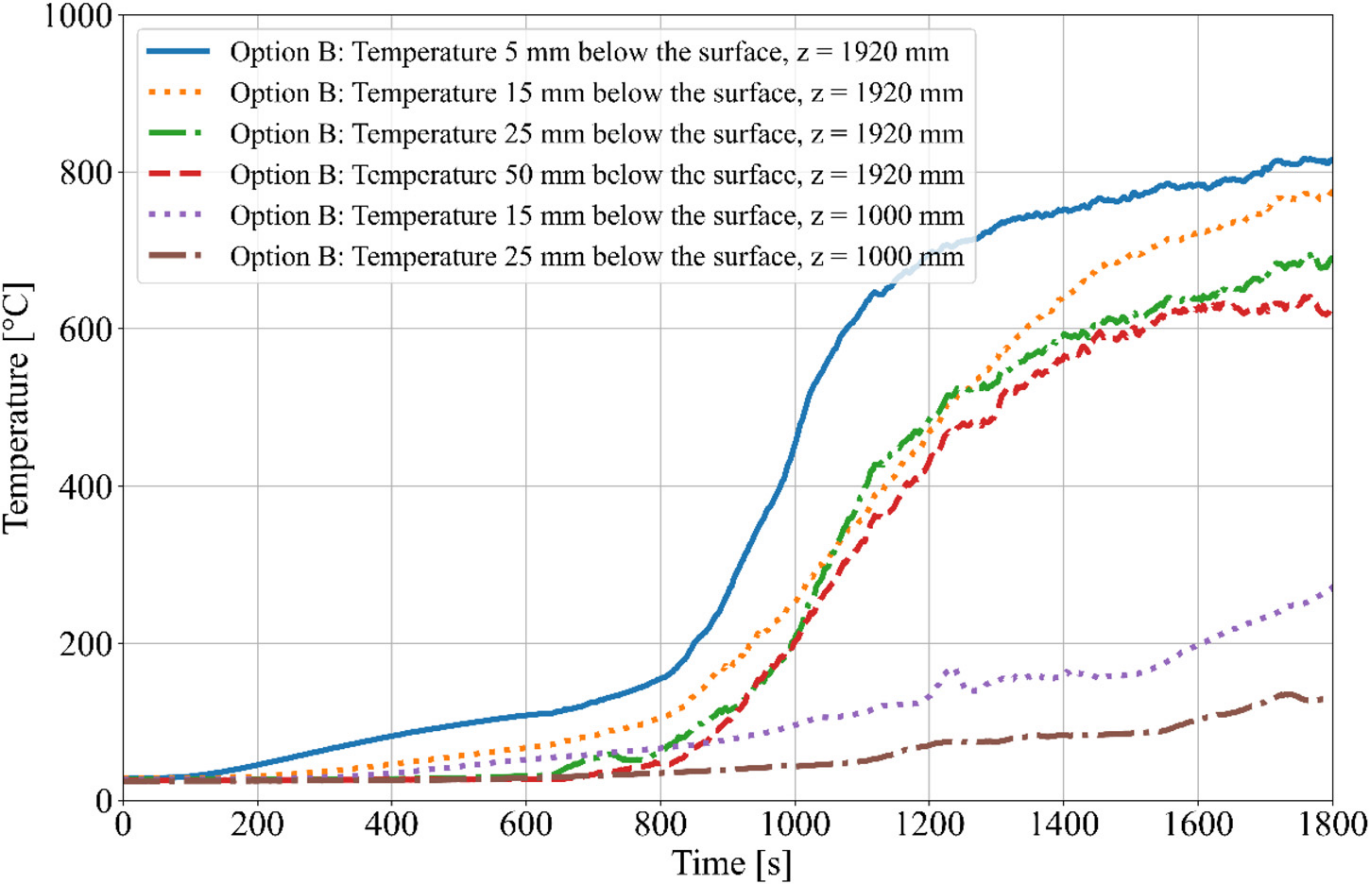
Option B - Column 1: temperature.

The burner performance and temperature evolution within the RCT furnace were designed to simulate the growth phase of a natural fire. Temperature data from Column 1 are presented in Fig. 14 for Option A and Fig. 15 for Option B. The focus on Column 1 is due to its critical proximity to the fire source, making it the most relevant for further evaluation of the charred layer development. Experimental results indicate that Option B exhibited higher temperatures compared to Option A, which is attributed to the different spatial arrangement of the timber members and the resulting thermal exposure.

**Fig. 16:**
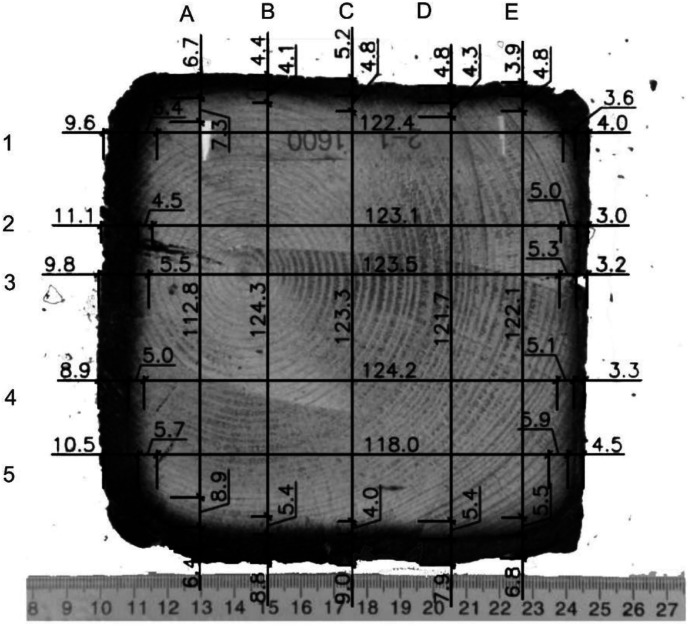
Method of measuring the charred layer after the experiment.

**Fig. 17:**
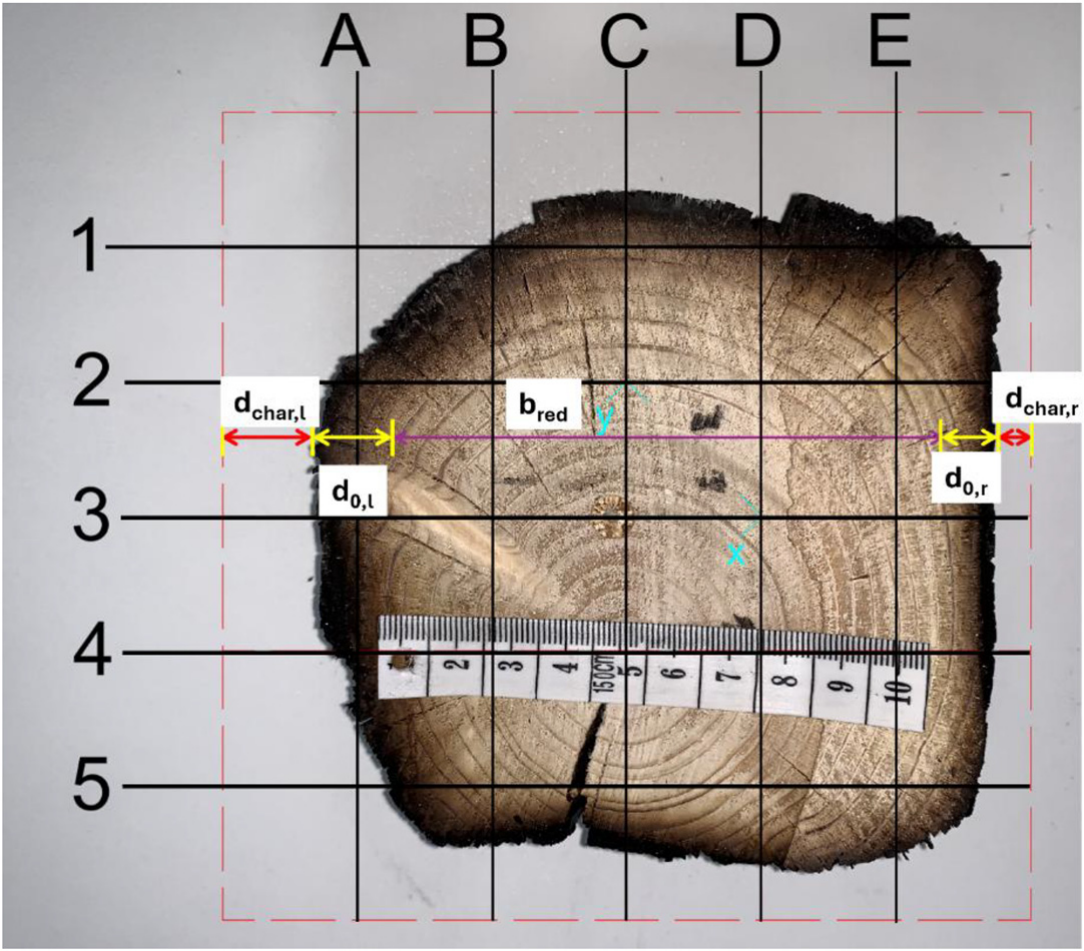
Drawing of the symbols used.

The depth of the charred layer was determined from photographs of individual cross-sections of the tested timber elements. After cooling in a water bath, the columns and beams were cut perpendicularly to their axis to expose their internal structure. The spacing and frequency of the cuts varied depending on the charring rate and fire exposure; however, the average distance between cuts was 20 or 50 mm. Each section was numbered, described, and carefully positioned horizontally before being photographed with a scale reference. The camera was centered to ensure maximum comparability of the cross-sectional dimensions across different specimens. These images were then processed in software, where the charred layer, pyrolysis layer, and reduced cross-section were measured (see Fig. 16, Fig. 17).

For systematic photo documentation, a fixed stand was used to position a camera at a consistent distance from each sample, ensuring uniform image scaling across all sections. This approach was necessary due to the large number of cross-sections requiring measurement. The cut samples were placed on a white background to enhance the visibility of charred layers. Most images were taken at a known scale, allowing verification of measurement consistency. However, due to non-uniform charred cross-section geometry, it was not always possible to ensure the identical positioning of specimens. To mitigate this, one identical side of the second measured column was consistently marked, ensuring that all images maintained the same orientation. Although fire exposure made it visually clear which sides had been exposed to flames, marking the reference side facilitated a more reliable comparison of different elements, particularly when aligning cross-sections during analysis.

After photo documentation, all images were uploaded into AutoCAD software, where they were rescaled using the reference scale embedded in the photographs. Charring depth, pyrolysis layer thickness, and reduced cross-section dimensions were then measured using a grid system with 25 mm increments. The boundaries between the charred layer, pyrolysis layer, and unburned wood were determined through visual assessment. However, distinguishing the transition between pyrolyzed and unburned wood presents a challenge, as the process occurs gradually, resulting in a continuous color transition rather than a sharp boundary. Consequently, measurement uncertainties must be taken into account. To improve accuracy, advanced techniques such as 3D scanning and automated photographic analysis are recommended.

For sections where the charred layer was more developed, accuracy of the measurement had the greatest impact on description of charred layer thickness. To capture its variability, measurements were conducted along multiple axes visible at (Fig. 16, Fig. 17). The recorded values for Option A are presented in Fig. 24, Fig. 25, while the data for Option B are shown in Fig. 26, Fig. 27.

Additional measurement biases may have resulted from corner rounding, as the corners were not measured radially but only along specific measurement axes. A comparative analysis of Column 1 at different heights is shown in Fig. 28. In Fig. 27, the missing data points in the middle section of the graph indicate that measurements were not possible in areas where the column had burned below the depth of the section at that location.

It is important to note that the reported charred layer represent the final state after 30 min of fire exposure. In Option B, a clear increase in charring rate at the top of Column 1 was observed, which can be attributed to higher gas temperatures in the upper smoke layer. In contrast, in the Option A, the difference in charring depth between the bottom and top of the column was less pronounced, with variations in the range of only a few millimeters.

The non-uniform charring depth along the column height indicates that charring is significantly influenced by temperature gradients and gas flow dynamics. Additionally, a notable difference in charring between the first and second experiments was observed, suggesting that factors such as burner power, heat flux, and fuel combustion rate play a crucial role.

Another key factor affecting charring development is the orientation of the timber member relative to the fire source. This aspect was analyzed primarily on Column 1, which was positioned closest to the burner. Two of its sides were directly exposed to the radiative part of heat flux from the flames, whereas the remaining two sides faced away, experiencing lower thermal exposure coming from convective heat flux mainly.

In Option A, the sides exposed to the fire up to a height of approximately 1300 mm remained largely uncharred. The charring layer only started forming between 1300 and 1400 mm, where the intensity of fire exposure increased. At this height, the measured charring depths varied, with 7.9 mm on the left side, 7.3 mm on the right side, 9.1 mm on the upper side, and 4.4 mm on the lower side. Since the column was symmetrically exposed along the diagonal axis, the expected char depth should have been similar for the bottom and left sides. However, the bottom side exhibited a thinner char layer, likely due to the positioning of the raster B axis, which was located closer to the fire. This positioning may have contributed to higher local turbulence and more pronounced erosion of the char layer, reducing its thickness.

In Option B, where burner power was increased, all three timber columns ignited almost simultaneously across their entire surface area, leading to a higher overall charring rate. The maximum recorded temperatures were at 1920 mm from the base of Column 1, which was the most severely charred and reached peak temperatures of approximately 820 °C. Column 2 ignited progressively from the top and side facing the burner, while Column 3 ignited sequentially from the top. Later in the test, flames from Column 1 spread to Column 3, contributing to further fire propagation.

**Fig. 18:**
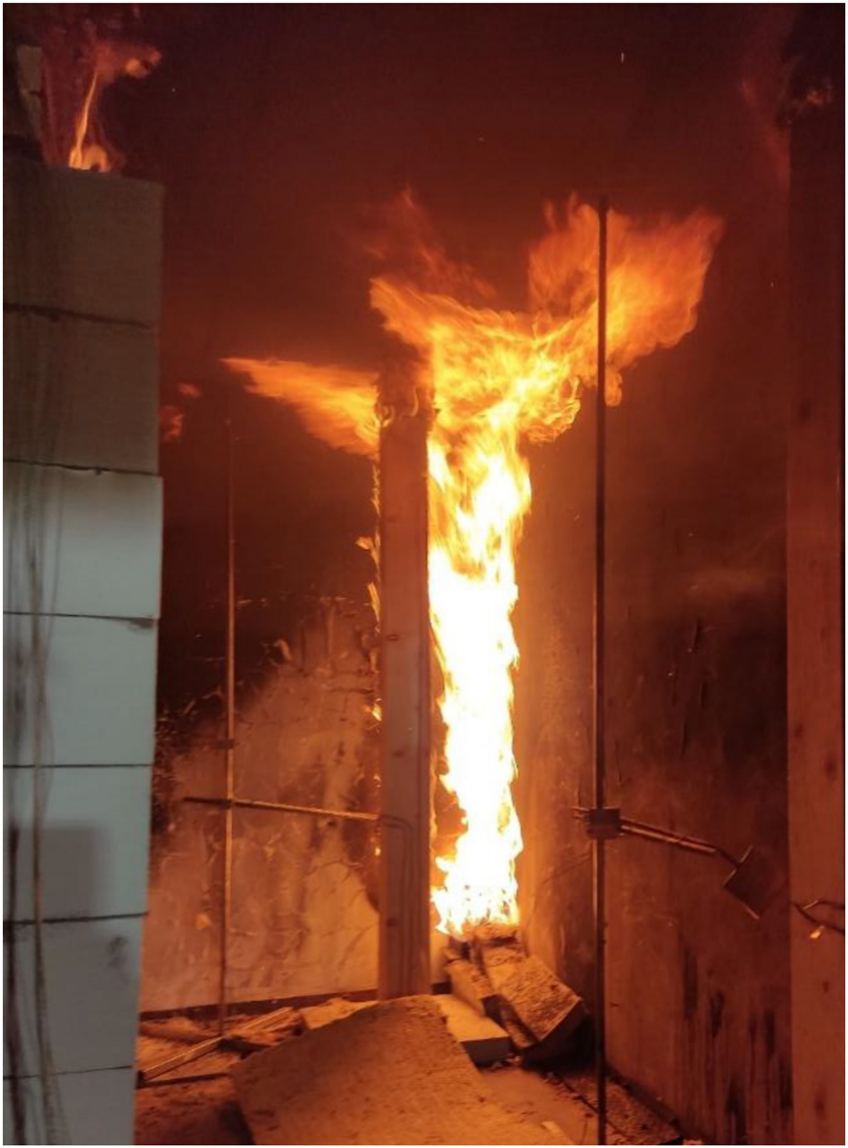
Experimental observations: Option A - view of the spread of flames along the column.

**Fig. 19:**
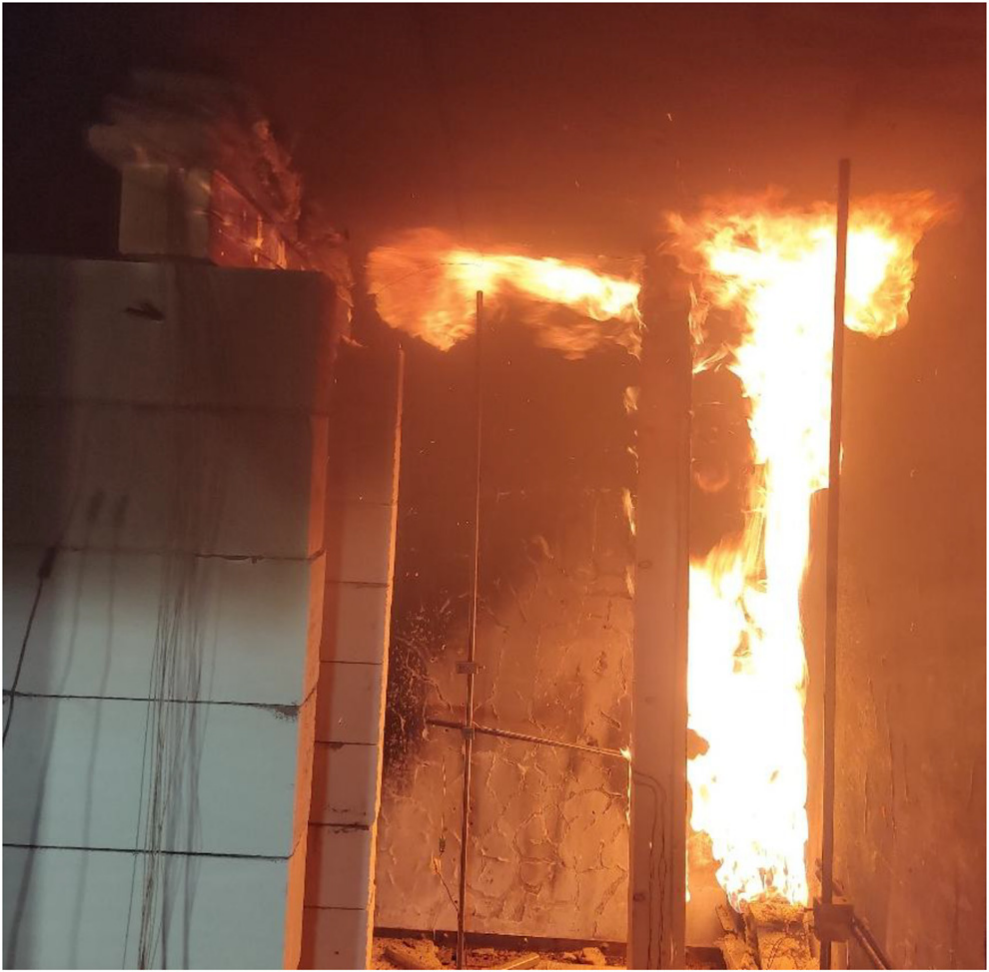
Experimental observations: Option A - view of the spread of flames along the beam.

**Fig. 20:**
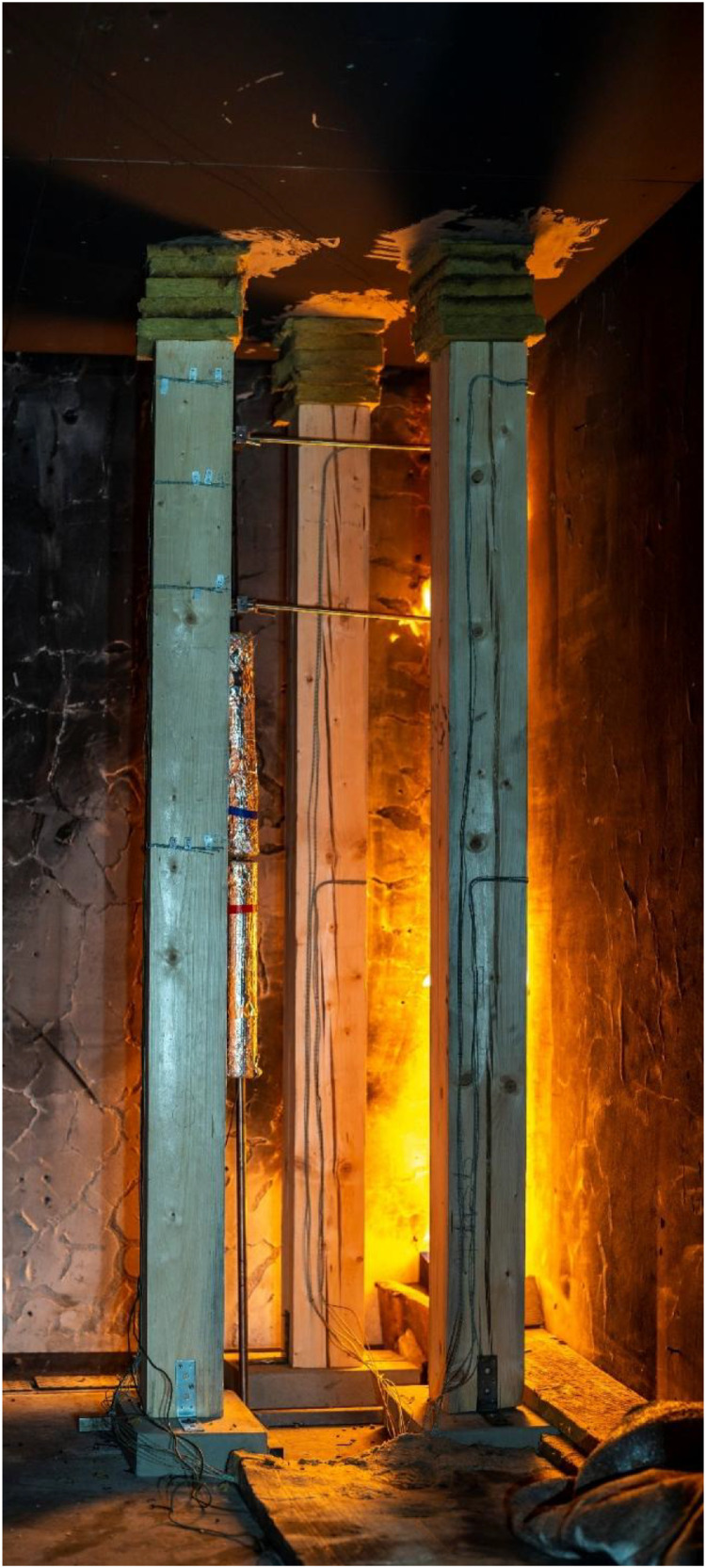
Experimental observations: Option B - start of experiment.

**Fig. 21:**
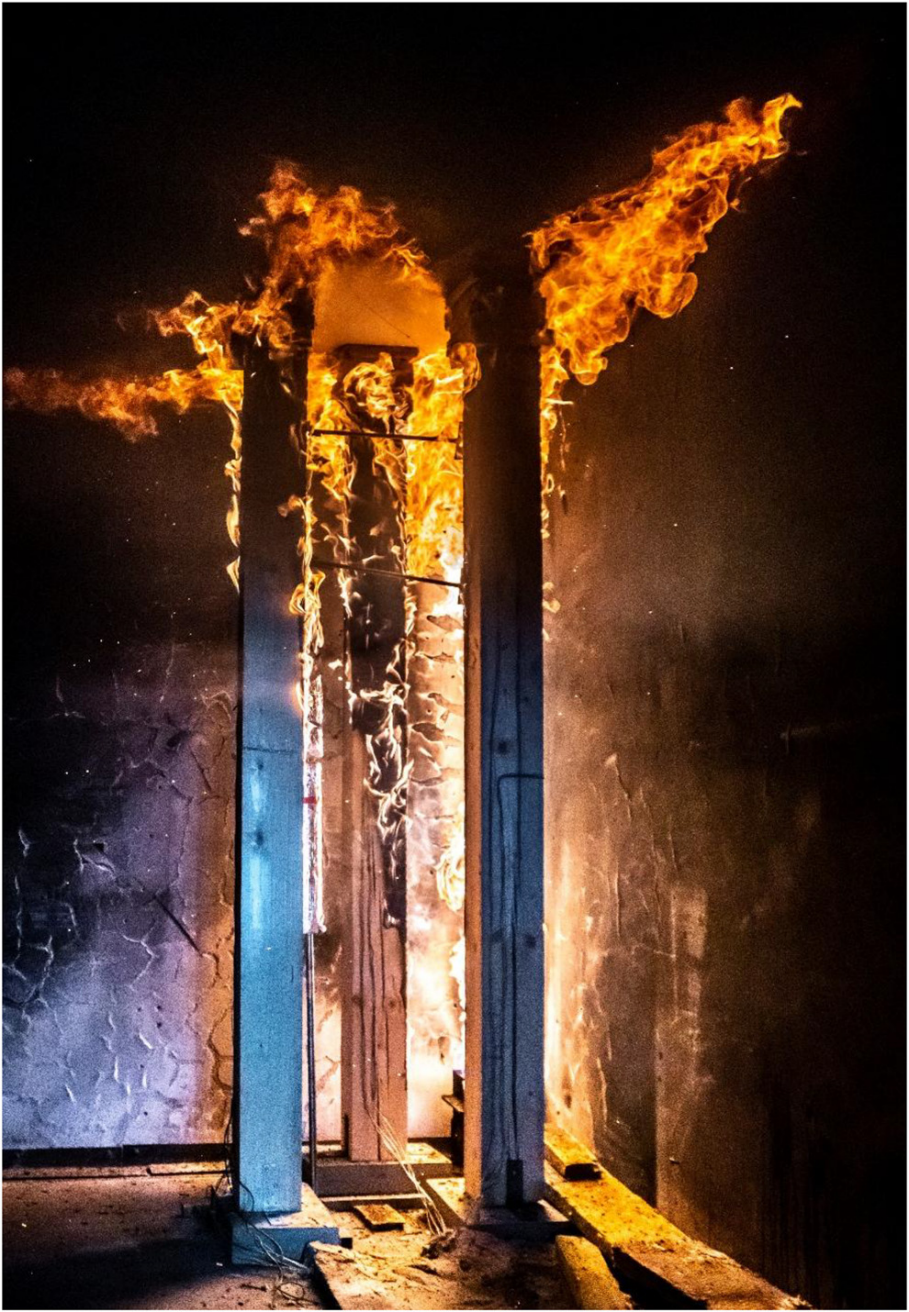
Experimental observations: Option B - view of the spread of flames along the.

**Fig. 22:**
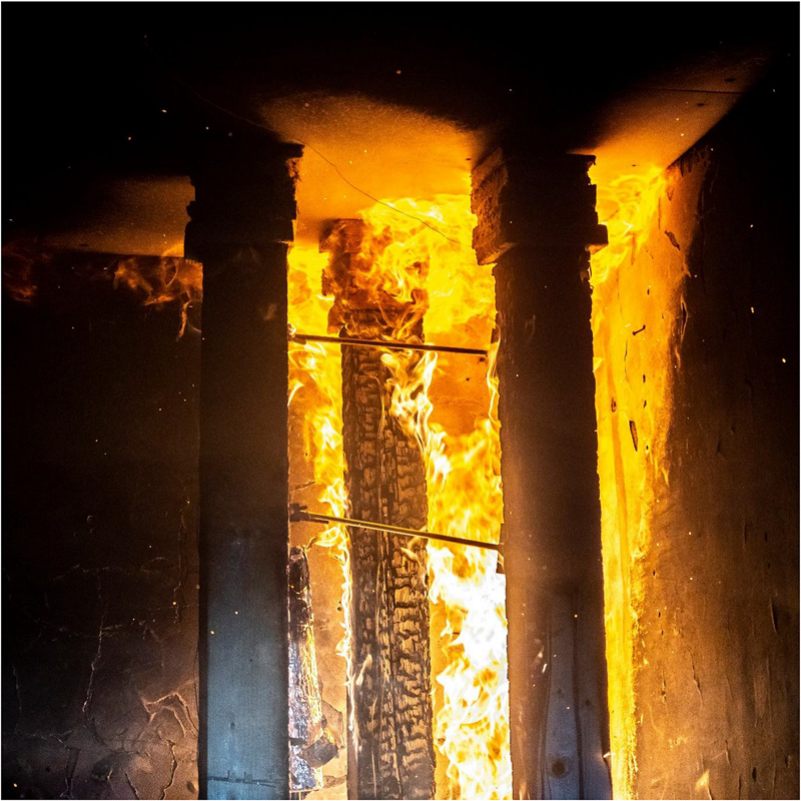
Experimental observations: Option B - close-up of the shape of the flames.

**Fig. 23:**
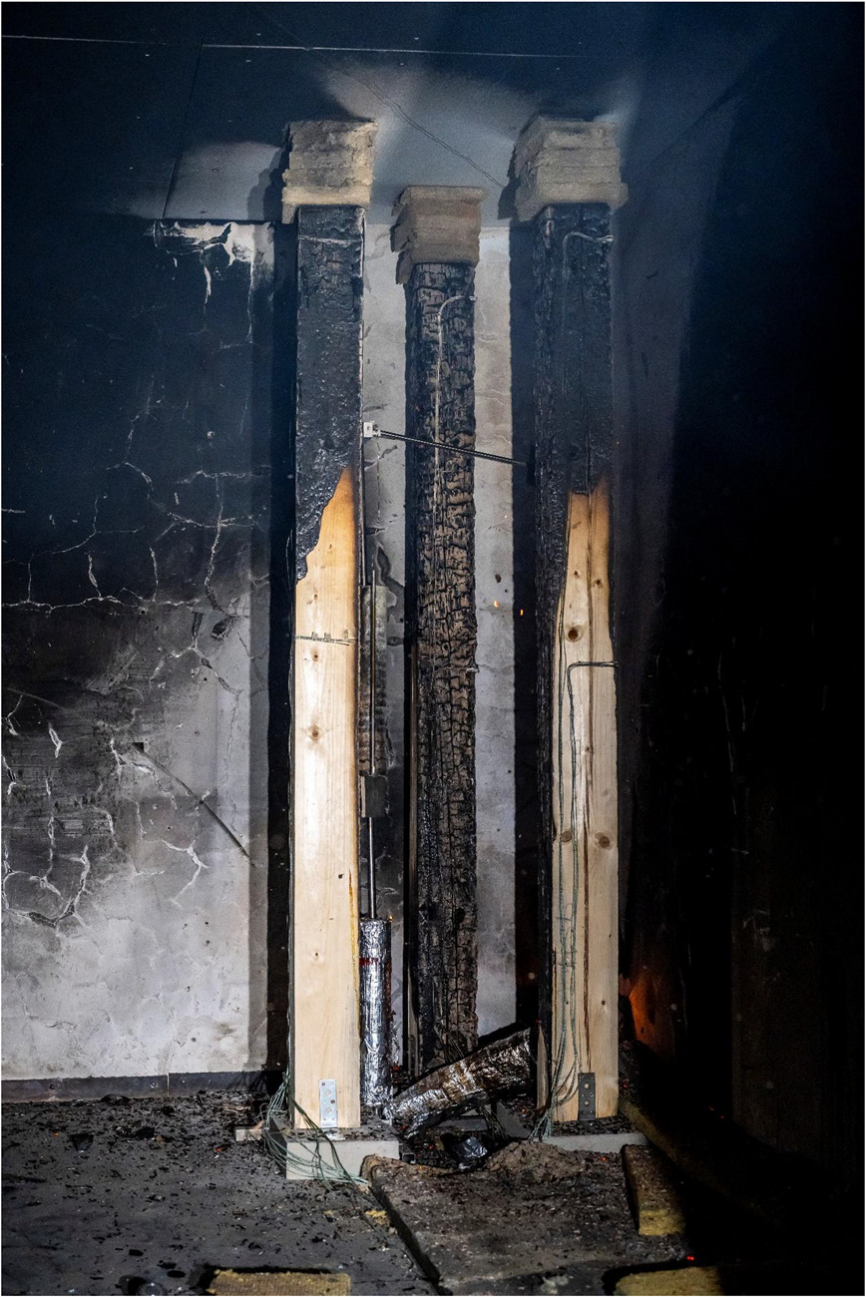
Experimental observations: Option B - condition of the columns after the experiment.

Results of the experiments demonstrate the combined effects of sample positioning, orientation relative to the fire source, and burner power on timber combustion and charring development. Additionally, material defects such as cracks and knots play a role in charring variability, as cracks facilitate deeper oxygen penetration, accelerating combustion within these weakened regions. Such defects are difficult to systematically account for in predictive models but represent a relevant factor in real fire scenarios. Experimental observations for Option A are documented in Fig. 18, Fig. 19, while those for Option B are shown in Fig. 20, Fig. 21, Fig. 22, Fig. 23.

**Fig. 24:**
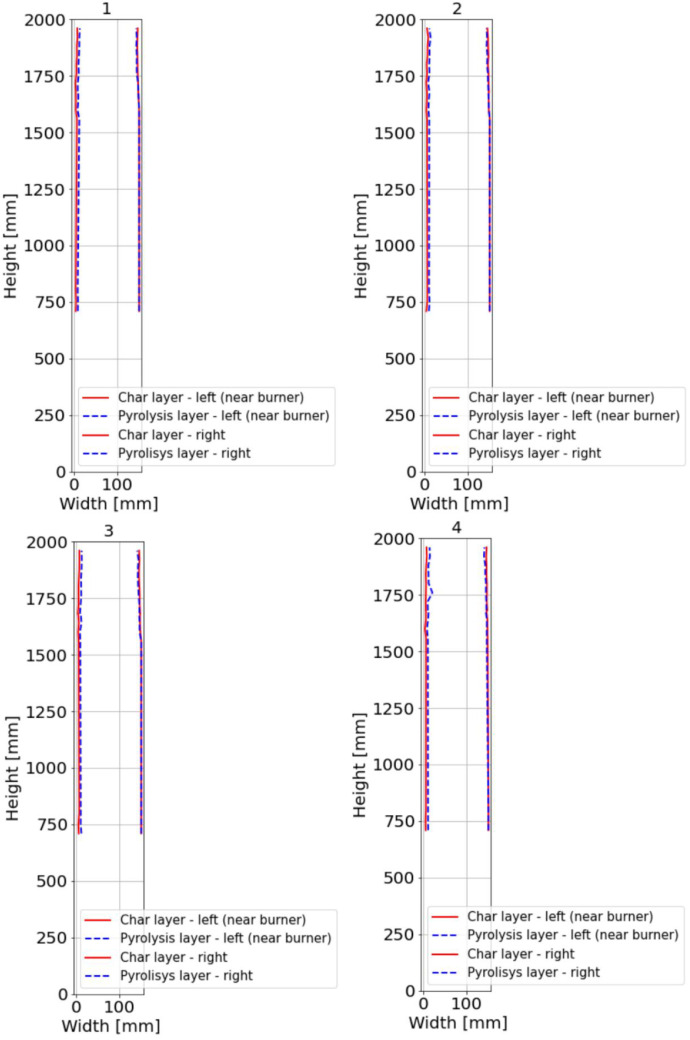
Option A: Plotted development of the charred layer and pyrolysis layer, Sections 1 to 4.

**Fig. 25:**
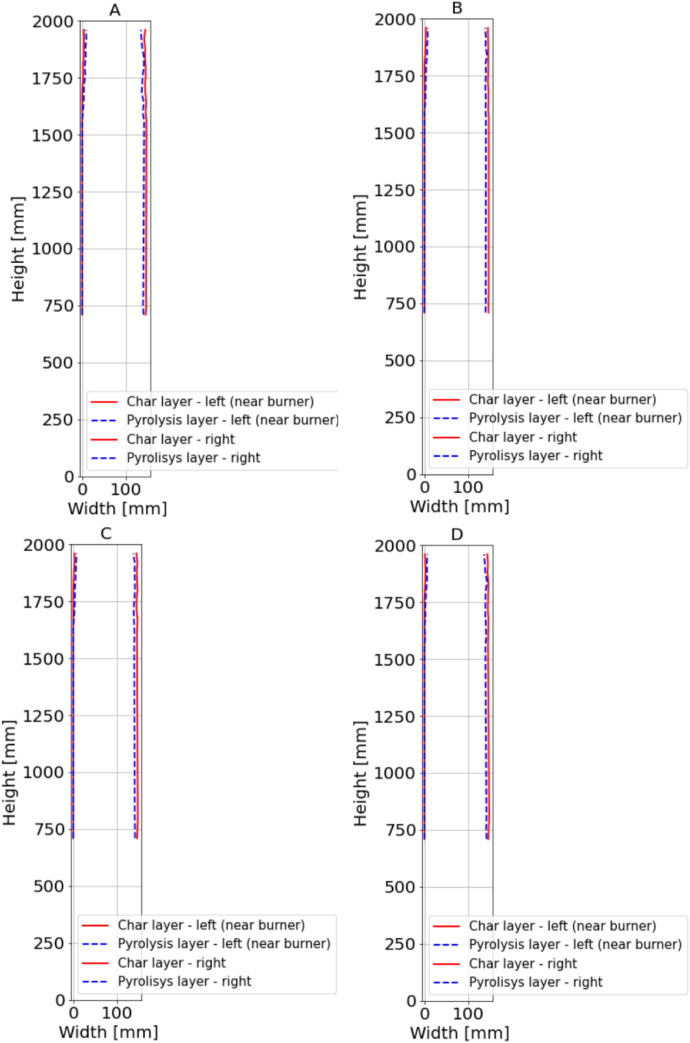
Option A: Plotted development of the charred layer and pyrolysis layer, Sections A to D.

**Fig. 26:**
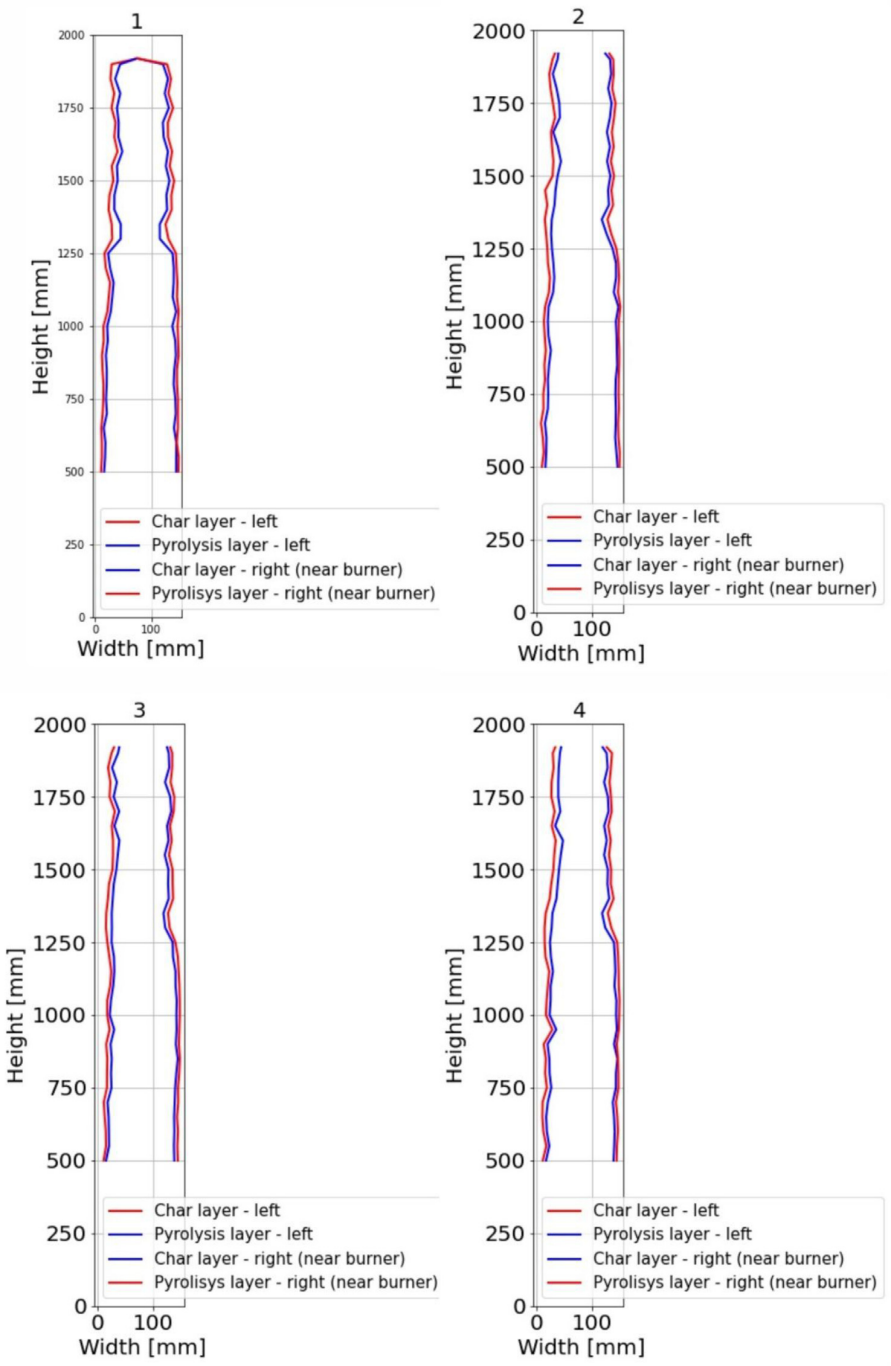
Option B: Plotted development of the charred layer and pyrolysis layer, Sections 1 to 4.

**Fig. 27:**
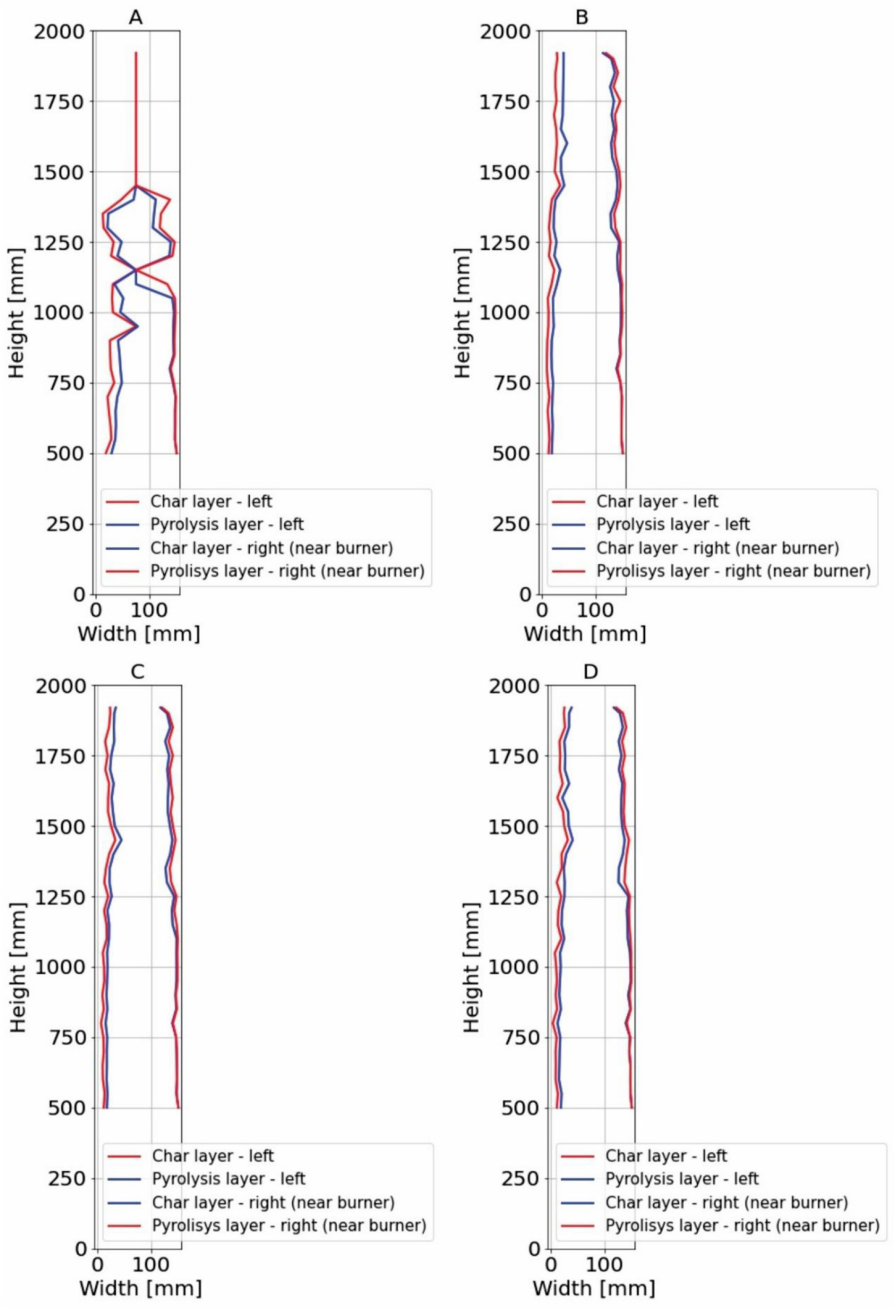
Option B: Plotted development of the charred layer and pyrolysis layer, Sections A to D.

**Fig. 28:**
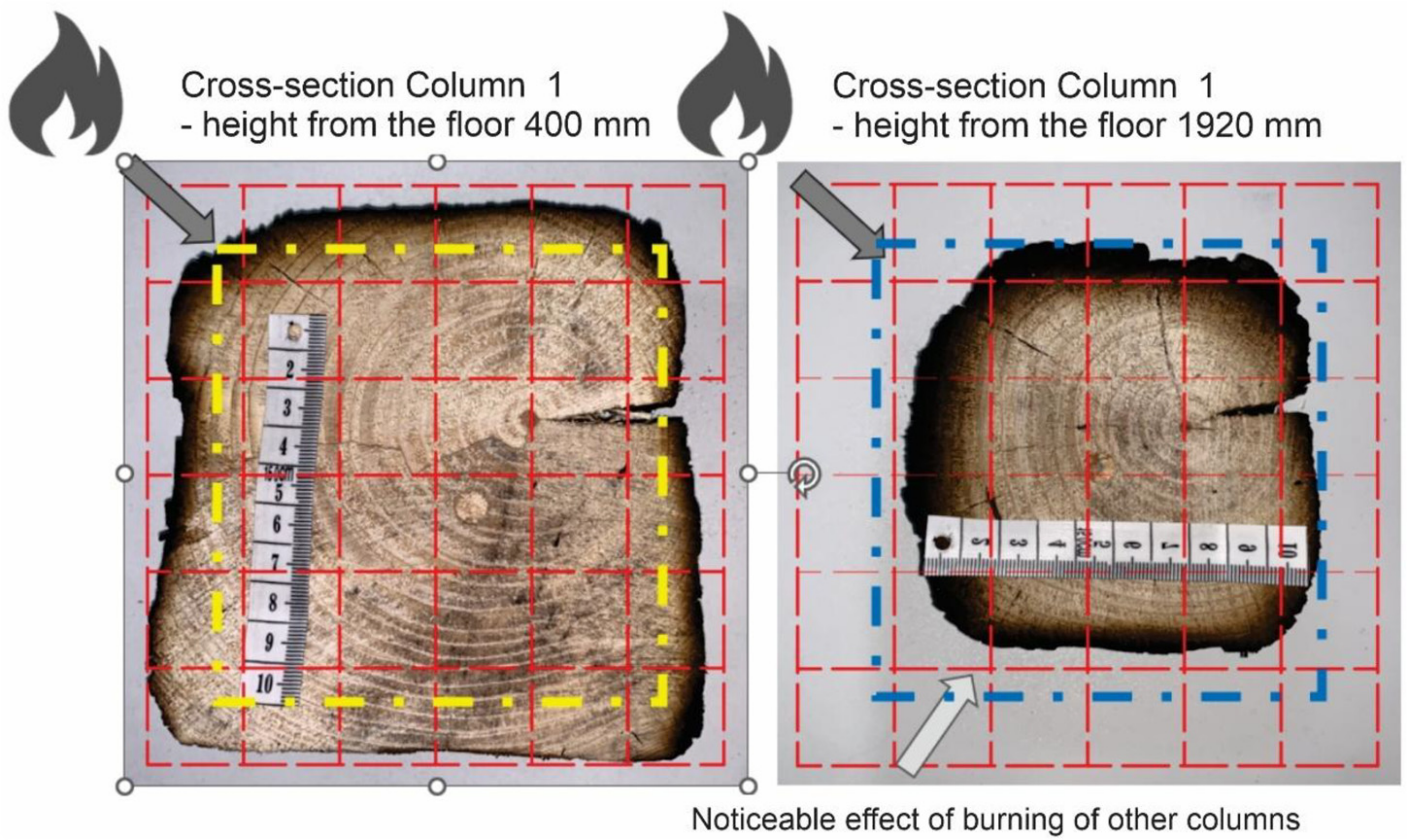
Option B: Comparison of the depth of charring on Column 1 at heights of 400 and 1920 mm.

The position of the 300 °C isotherm was determined from measured temperature data. For Column 1 in Option A, this isotherm corresponded to a char depth of 16.5 mm at a height of 1920 mm, which closely matched the measured charred layer thickness of 16.3 mm at 1960 mm. This suggests that the 300 °C isotherm could approximate the boundary of the charred layer, though it must be noted that this correlation was observed only for a 30 min test duration. Based on the maximum recorded charring depth in Column 1 (Option A), a charring rate of 0.54 mm/min was calculated. This value suggests that the charring rate may be more conservative than the standardized approach. However, it is important to recognize that charring rates are not constant over time, particularly under natural fire conditions, and the final charring depth observed in this study was 15 mm less than the standard prediction.

## Limitations

The dataset is based on experimental fire tests conducted under controlled laboratory conditions, which may not fully capture the variability present in real-scale fire scenarios. Minor uncertainties may arise from measurement tolerances of thermocouples and heat flux sensors, as well as from the repeatability of material preparation and mounting during testing. While all instrumentation was regularly calibrated and verified, slight deviations in boundary conditions (such as ventilation rate or local flame impingement) could influence temperature development at specific locations. The dataset represents a well-documented and internally consistent collection of results suitable for model validation and comparative studies; however, its applicability to highly variable real fires should be considered with appropriate engineering judgment.

## Ethics Statement

The authors confirm that they have read and follow the ethical requirements for publication in Data in Brief. The current work does not involve human subjects, animal experiments, or any data collected from social media platforms. All experiments were conducted in accordance with the safety regulations of the fire testing laboratories of the Czech Technical University in Prague, ensuring full compliance with institutional safety procedures and risk management guidelines.

## CRediT Author Statement

**Jakub Šejna:** Conceptualization, Methodology, Investigation, Data Curation, Writing – Original Draft, Visualization, Supervision **Dominik Štraus:** Formal Analysis, Validation, Visualization **Marie Křišťanová:** Formal Analysis, Validation, Visualization **Simona Rušarová:** Resources, Data Curation, Writing – Review & Editing **Kamila Cábová:** Resources, Data Curation, Writing – Review & Editing. **Robert Pečenko:** Writing – Review & Editing **Tomaž Hozjan:** Writing – Review & Editing **František Wald:** Project administration, Funding acquisition.

## Data Availability

ZenodoExperimental dataset on the charring behavior of timber elements under natural fire conditions (Original data).
